# MiR-210-3p protects endometriotic cells from oxidative stress-induced cell cycle arrest by targeting BARD1

**DOI:** 10.1038/s41419-019-1395-6

**Published:** 2019-02-13

**Authors:** Yongdong Dai, Xiang Lin, Wenzhi Xu, Xiaona Lin, Qianmeng Huang, Libing Shi, Yibin Pan, Yinli Zhang, Yunshan Zhu, Chao Li, Lulu Liu, Songying Zhang

**Affiliations:** 1 Assisted Reproduction Unit, Department of Obstetrics and Gynecology, Sir Run Run Shaw Hospital, Zhejiang University School of Medicine, No. 3 Qingchun East Road, Jianggan District, 310016 Hangzhou, China; 2 Key Laboratory of Reproductive Dysfunction Management of Zhejiang Province, No. 3 Qingchun East Road, Jianggan District, 310016 Hangzhou, China; 3 Cancer Biotherapy Center, The First Affiliated Hospital, Zhejiang University School of Medicine, No. 79 Qingchun Road, Xiacheng District, 310003 Hangzhou, China

## Abstract

Endometriosis is associated with benign but adversely developed cysts in the extrauterine environment. The oxidative imbalanced environment induces DNA damage and affects cell cycle progression of endometrial stromal cells (ESCs) and endometrial epithelial cells, but how endometriotic cells maintain proliferation in the presence of oxidative stress is not clear. Growing evidence has indicated that the ectopic hypoxic microenvironment and oxidative stress can stimulate the growth of endometriotic cells, which is mainly due to the increase of HIF-1α. We found that the master hypoxia-associated miRNA miR-210-3p was increased in stromal and glandular cells of ectopic lesions compared with that of eutopic and normal endometria and was consistent with the expression of HIF-1α and the local oxidative stress-induced DNA damage predictor 8-OHdG. Moreover, miR-210-3p was upregulated in ESCs and Ishikawa cells under hypoxic conditions but not in normoxic culture. Knockdown of miR-210-3p induced a G2/M arrest of ESCs and Ishikawa cells under hypoxia, while no effect was found under normoxia. BARD1 was identified as a target of miR-210-3p. BARD1 expression was decreased in endometriotic tissues compared with eutopic and normal endometria and negatively correlated with the expression of miR-210-3p. Multivariate regression analysis showed that BARD1 downregulation could serve as an indicator for endometriotic severity. Our results suggest that miR-210-3p attenuates the G2/M cell cycle checkpoint by inactivating BRCA1 complex function in response to DNA damage under hypoxia via targeting the 3′ untranslated region of BARD1 mRNA. Endometriotic mouse model experiments showed that intraperitoneal injection of the miR-210-3p inhibitor or vitamin C suppressed the growth of endometriotic lesions. Together, our results demonstrate that endometriotic cells inhibit BARD1/BRCA1 function by upregulating miR-210-3p, which might be the underlying mechanism for endometriotic cell maintenance of growth in oxidative stress. Furthermore, inhibition of miR-210-3p and administration of vitamin C are promising approaches for the treatment of endometriosis.

## Introduction

Endometriosis is a common oestrogen-dependent gynaecologic disease that is defined as the proliferation of endometrial-like tissue outside the uterus cavity. Endometriosis is one of the main causes of infertility in reproductive aged women^1^. Recent studies have found that repeated cyclical haemorrhage is involved in the initiation and progression of endometriosis via inducing excessive oxidative stress (OS)^2^, which is defined as an imbalance between reactive oxygen species (ROS) and antioxidants^3,4^. Many studies on OS-associated diseases suggest that oxidative balance is complicated and precarious^5^, as ROS not only modifies proteins, impacts lipids, damages DNA strand structure and regulates cell cycle checkpoints^6,7^, but also maintains survival, intensifies adhesion, promotes angiogenesis and facilitates cell cycle progression^8–10^. In endometriosis, excessive OS results in higher DNA damage and reduced DNA repair activity^3,11^. However, the mechanisms by which adverse molecular alterations, such as excessive ROS, induce the DNA damage repair response in endometriotic cells, which show continuous cell cycle progression, is obscure.

Endometriotic tissues show increased levels of hypoxia, which is believed to stimulate the establishment of ectopic lesions via enhancement of adhesion, angiogenesis and proliferation^12–15^. Intriguingly, excessive ROS in endometriosis stimulates the expression of hypoxia-inducible factor 1α (HIF-1α)^16,17^, the key regulator of hypoxia. Moreover, ROS and HIF-1α have a reciprocal inductive relationship under hypoxia^18^, as stabilisation of HIF-1α under hypoxia requires generation of ROS from the Qo site of mitochondrial complex III^19,20^, and HIF-1α initially triggers ROS expression by inhibiting the mitochondrial electron transport chain at complex I or activating NADPH oxidase;^21,22^ activated HIF-1α then aggravates ROS production via increasing pro-oxidants or decreasing antioxidants^18,23^. Although the positive feedback regulation between ROS and HIF-1α has been proven in many different diseases, their specific interaction in endometriosis has not been determined.

MicroRNAs (miRNAs) function by binding specific seed sequences in the 3′-untranslated region (3′-UTR) of target mRNAs, which results in translational inhibition, mRNA degradation or mRNA destabilisation^24^. Several hypoxia-associated miRNAs have been found directly target genes involved in survival, proliferation, migration and metabolism of endometriotic cells^25–27^. MiR-210-3p is a master HIF-1α-responsive hypoxia-associated miRNA that is highly expressed in endometriosis and stimulates cell proliferation via activating STAT3^28,29^. However, current studies have been restricted to the putative mechanisms linking miR-210 and endometriosis development, and little is known about the potential regulatory functions and downstream targets of miR-210-3p in endometriotic lesions.

As hypoxia and ROS play important roles in endometriosis and based on their functional connections in other diseases, we speculated that hypoxia-associated miR-210-3p and ROS-triggered DNA damage may be linked in endometriotic lesions. Furthermore, how endometriotic cells maintain proliferation under hypoxic conditions that risk DNA damage has remained unclear. Here we examined the relationship between hypoxia and DNA damage in endometriosis and explored the function of miR-210-3p and its downstream targets in endometriotic cells.

## Materials and methods

### Study approval

This study was initiated on 23 December 2016 and terminated on 14 July 2018. The study was approved and monitored by the ethics committee of Sir Run Run Shaw Hospital, Zhejiang University. Informed written consent was obtained from each patient before tissue collection. All animals were maintained in accordance with the National Institutes of Health Guide for the Care and Use of Laboratory Animals, and the experiments were approved by the Committee of Experimental Animal Ethics, Zhejiang University.

### Specimen collection, primary endometrial stromal cell extraction and authentication

Paired eutopic endometria and ectopic lesions from reproductive-aged women with deep infiltrating endometriosis were collected. Endometrial stromal cells (ESCs) were purified from primary cultured cells of eutopic endometria as previously described^15^. Immunofluorescense was performed to determine the purity of isolated ESCs by staining human CD10 and Pan-cytokeratin (results showed in Supplementary Figure 1). The human endometriosis-derived immortalised eutopic endometrium stromal cell line hEM15A^30^ and human endometrial carcinoma cell line Ishikawa were purchased from BeNa Culture Collection (BNCC 340223 and BNCC 338693, Kunshan, China). Cells were cultured at 37 °C in 21% O_2_ as normoxic culture and 1% O_2_ as hypoxic culture. Hypoxic conditions were maintained using a Hypoxia Incubator Chamber (27310, STEMCELL, Canada) with 5% CO_2_ and 1% O_2_ balanced with N_2_. Detailed inclusion criteria for patients, numbers of cases and primary cell culture information are presented in the Supplementary Information; the clinical characteristics of patients are summarised in Supplementary Table 1.

### Immunohistochemistry

Immunohistochemistry was performed according to standard procedures. A semi-quantitative grading system (*H*-score) was used to evaluate the intensity and percentage of staining. *H*-score of BARD1 in ectopic lesions that within 95% confidence interval of its expression in normal endometria (95% CI) was defined as BARD1 positive, while those less than 95% CI was defined as BARD1 negative. The primary antibodies are listed in Supplementary Table 2. Detailed procedures are provided in the Supplementary Information.

### Alkaline comet assay

Cells were cultured under hypoxic conditions for 28 days and alkaline comet assays were performed following the protocol provided by Trevigen (Gaithersburg, MD, USA). Detailed procedures are provided in the Supplementary Information.

### High-throughput sequencing

ESCs were collected after hypoxic or normoxic culture for 48 h and then sent to RiboBio (RiboBio Co. Ltd, Guangzhou, China) for miRNA high-throughput sequencing analysis. RNA high-throughput sequencing was conducted to compared the global gene expression profile between miR-210-3p-overexpressing hEM15A cells and miRNA control-expressing cells. Only miRNAs or transcripts with more than two-fold changes and a *P* value less than 0.05 were considered statistically significant.

### RNA isolation, quantitative reverse transcriptase PCR and western blot

RNA isolation, quantitative reverse transcriptase PCR (qRT-PCR) and western blot assay were performed according to standard procedures. Detailed procedures are listed in the Supplementary Information. Primers are listed in Supplementary Table 3.

### Fluorescence in situ hybridisation analysis

Fluorescence in situ hybridisation (FISH) analysis was performed in five normal endometrium and five paired eutopic and ectopic tissues according to standard methods^31^. An oligonucleotide probe complementary to miR-210-3p was purchased from Guangzhou Exon Biotechnology Co. Ltd (Guangzhou, China). Immunofluorescent double staining of CD10 and Pan-cytokeratin was used to define the location of miR-210-3p.

### Lentivirus and infection

MiR-210-3p overexpression lentivirus (abbreviated as LV-210), miR-210-3p short hairpin RNA lentivirus (LV-In-210), corresponding negative control lentiviruses (LV-CN or LV-In-CN), BARD1 overexpression lentivirus (LV-BARD1) and BARD1 short hairpin RNA lentivirus (LV-In-BARD1) were purchased from Genechem Co., Ltd (Shanghai, China). Cells were infected with lentiviruses at 10 MOI (multiplicity of infection) for 48 h in normoxic or hypoxic conditions in parallel.

### Luciferase assay

For luciferase assays, 293 T cells were transfected with luciferase reporter plasmids and miR-210-3p mimic or controls using Lipofectamine 3000 (Invitrogen, Carlsbad, CA, USA). Luciferase activity was analysed using a Dual-Luciferase® Reporter Assay System (Promega, Madison, WI, USA). Detailed procedures are provided in the Supplementary Information.

### Flow cytometry assay

Cells infected with lentivirus were fixed and stained with propidium iodide by standard protocols and then analysed using a FACSCalibur (BD, CA, USA). Data were analysed using Mod Fit LT 3.0 software. Detailed procedures are described in the Supplementary Information.

### Establishment of the endometriotic mouse model

Endometriosis was induced in 16 C57BL6 mice by mouse–mouse intraperitoneal implantation as described previously^32^. MiR-210-3p inhibitor (abbreviated as In-210) or its negative control (abbreviated as NC) was intraperitoneally injected in endometriotic mice (*n* = 8, each group) using the in vivo-jet PEI delivery agent (Poly Plus, NY, USA).

For vitamin C experiments, another 33 endometriotic C57BL/6 mice were established. Eleven endometriotic mice were given 2.5 mg vitamin C in 1 mL purified water via oral gavage each day^33^, 11 endometriotic mice received intraperitoneal injection of vitamin C (500 mg/kg) every other day^34,35^ and 11 endometriotic mice received intraperitoneal injection of phosphate-buffered saline (PBS).

Animals were sacrificed after 4 weeks, and endometriotic cysts were excised. The volumes of endometriotic cysts were calculated as follows: *V* (volume) = *LW*^2^/2, where *L* represents the largest length and *W* represents the smallest width. Detailed information is described in the Supplementary Information.

### Statistical analysis

SPSS program version 19.0, Graph Pad Prism 5 software was used for statistical analysis. Cell cycle and alkaline comet assay results were compared by paired Student *t-*test after confirming the normal distribution of the data by one-sample Kolmogorov–Smirnov test; variances in RNA and protein expression, cell cycle, cell proliferation, endometriotic cyst volume, *H*-scores and luciferase activities were compared by one-way ANOVA. Logistic regression analyses or chi square tests were used to assess the relationship between different clinical characteristics of patients and BARD1 expressions. Differences were considered to be statistically significant at *P* value ≤ 0.05.

## Results

### Endometriotic lesions show increased expression of HIF-1α and 8-OHdG

**Fig. 1:**
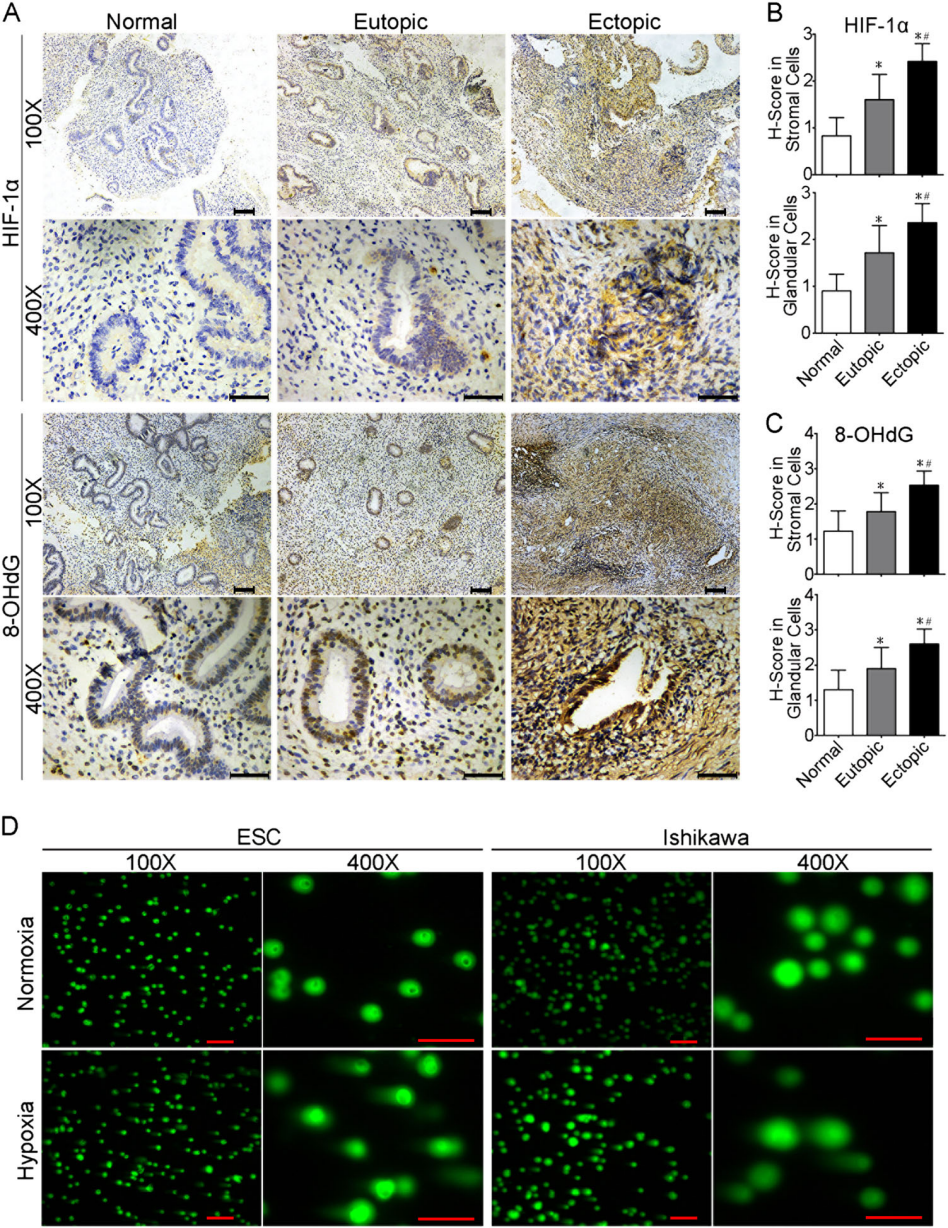
Higher reactive oxyben species (ROS) levels in ectopic lesions than eutopic and normal endometriums, and hypoxia induction of DNA damage in endometrial cells in vitro. **a** Immunohistochemistry photomicrographs of hypoxia-inducible factor 1α (HIF-1α) (the key regulator of hypoxia, which was stimulated by ROS) and 8-OHdG (an oxidative stress-induced DNA damage marker) in normal endometrium and paired eutopic and ectopic tissues. Scale bars = 100 µm for ×100 images. Scale bars = 50 µm for × 400 images. **b** Expression of HIF-1α in endometrial stromal cells (upper panel) and endometrial glandular cells (lower panel) by *H*-score (*y*-axis) was significantly higher in eutopic endometria and ectopic lesions than in normal endometria, with ectopic lesions showing the highest staining (*n* = 27 for normal endometria, *n* = 57 for paired eutopic and ectopic tissues). *P < 0.05 versus normal endometrial stromal cells; ^#^*P* < 0.05 versus eutopic endometrial stromal cells. One-way ANOVA with LSD for multiple comparisons. **c** Expression of 8-OHdG in endometrial stromal cells (upper panel) and endometrial glandular cells (lower panel) by *H*-score (*y*-axis) was significantly higher in eutopic endometria and ectopic lesions than in normal endometria, with ectopic lesions showing the highest staining (*n* = 27 for normal endometria, *n* = 57 for paired eutopic and ectopic tissues). *P < 0.05 versus normal endometrial stromal cells; ^#^*P* < 0.05 versus eutopic endometrial stromal cells. One-way ANOVA with LSD for multiple comparisons. **d** Representative fluorescence microscope images from alkaline comet assays of ESCs (left panel) and Ishikawa cells (right panel) after exposure to normoxia or hypoxia culture for 28 days. Scale bar = 200 μm for ×100 images. Scale bar = 100 μm for ×400 images. The tail lengths, tail DNA percentage and tail olive moments of comets were analysed using OpenComet. *n* = 5 for ESCs

Although it is reported that hypoxia leads to DNA damage and replication stress^36^, little is known about the relationship between hypoxia and DNA damage in endometriosis. We analysed the expression of HIF-1α, a key regulator of hypoxia, and 8-hydroxy-2-deoxyguanosine (8-OHdG), an OS-induced DNA damage marker, in normal endometria, eutopic endometria and ectopic lesions from endometriosis patients and healthy controls using immunohistochemistry (Fig. 1a). Baseline characteristics, such as body mass index, were similar between endometriosis patients and control individuals (Supplementary Table 1). HIF-1α and 8-OHdG expression in ESCs was significantly higher in eutopic endometria and ectopic lesions than in normal endometria, with ectopic lesions showing the strongest staining (Fig. 1b, c, *P* < 0.05, one-way ANOVA). HIF-1α and 8-OHdG expression in endometrial epithelial cells (EECs) showed a similar tendency (Fig. 1b, c, *P* < 0.05, one-way ANOVA). The same trend of HIF-1α and 8-OHdG expression in both stroma and gland implied that these two proteins may be involved in endometriosis progression.

### In vitro hypoxia culture induces DNA damage in ESCs and Ishikawa cells

We next investigated the effects of hypoxia on DNA damage by performing comet assays in ESCs and Ishikawa cells under normoxia or hypoxia. Hypoxia-cultured ESCs and Ishikawa showed significantly increased tail lengths, tail DNA percentage and tail olive moments compared with cells in normoxic culture, which indicated more severe DNA damage caused by hypoxia (Fig. 1d, Supplementary Figure 2, *P* < 0.05, Student's *t*-test). These results implied that hypoxia-associated molecules may facilitate cell growth in hypoxic conditions that risk DNA damage to endometrial cells.

### Hypoxic culture stimulates miR-210-3p expression in ESCs and Ishikawa cells

**Fig. 2:**
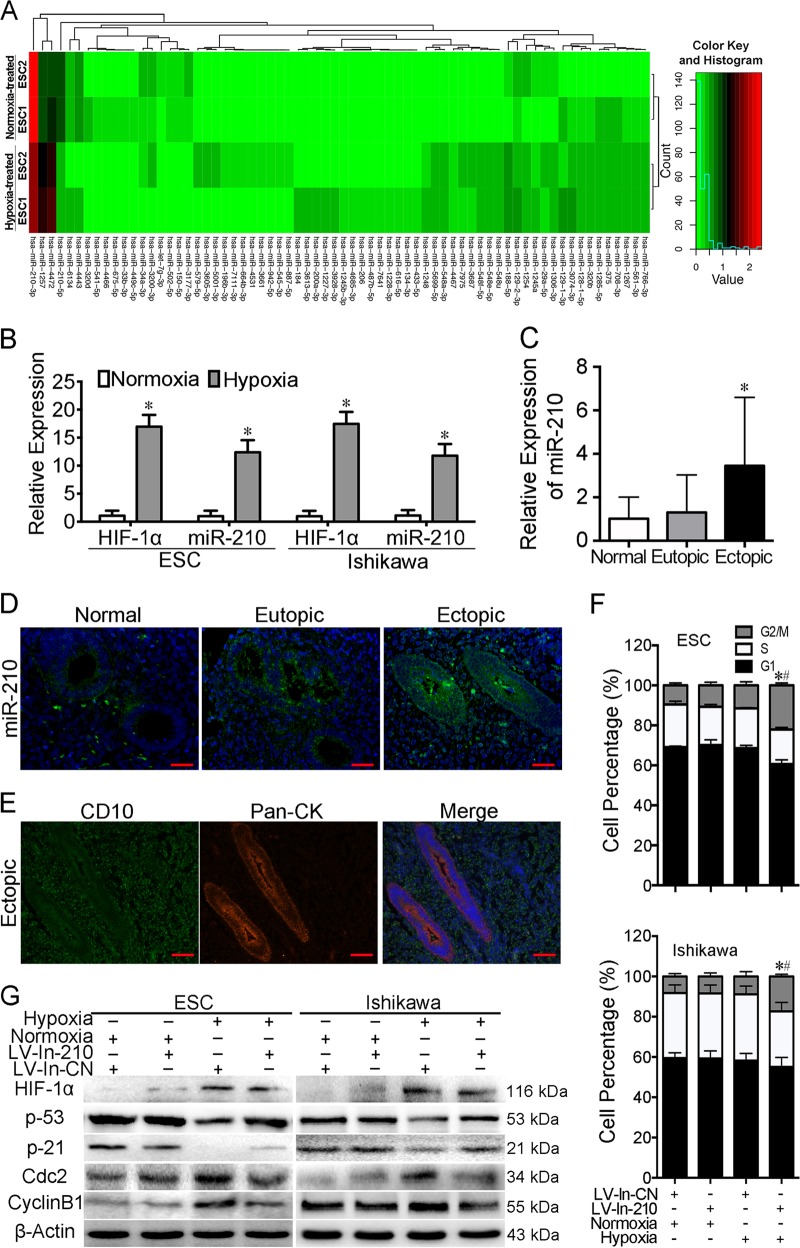
MiR-210-3p is upregulated in hypoxia and mediates G2/M arrest by hypoxia. **a** Differentially expressed miRNAs between normoxia and hypoxia pre-treated primary cultured endometrial stromal cells (ESCs) (*n* = 2) were determined by high-throughput sequencing and presented by the heat map. Each column represents one library, and the colour bar indicates relative expression level from high (red) to low (green). Red represents miRNAs showing a >2-fold change of expression; black represents miRNAs showing 1/2 < fold change ≤ 2; and green represents miRNAs showing a fold change ≤1/2. Hierarchical cluster analysis indicated that expression of miR-210-3p was high both in normoxia- and hypoxia-treated cells and was significantly upregulated after hypoxic treatment for 48 h. **b** Quantitative reverse transcriptase PCR (qRT-PCR) assay of miR-210-3p and HIF-1α mRNA expression in ESCs and Ishikawa cells cultured under normoxia or hypoxia. The bars show the mean value of three independent experiments ± standard deviation (*n* = 5 for ESCs). *P < 0.05 vs. normoxia, paired Student *t-*test. **c** qRT-PCR assay of miR-210-3p expression in human normal endometrium, paired eutopic endometrium and ectopic lesions (*n* = 15). *P < 0.05 vs. normal endometria, Mann–Whitney *U* test. **d** Fluorescence in situ hybridisation analyses using an oligonucleotide probe complementary to miR-210-3p on 5 μm sections of eutopic, ectopic endometrium and normal control tissues (*n* = 5). Scale bars = 50 μm. Original magnification: ×400. Blue represents DAPI; green represents miR-210-3p signal. **e** Representative immunofluorescence double staining images of ectopic lesion with anti-CD10 and anti-Pan-cytokeratin (Pan-CK) antibody. CD10, a stromal cell marker (green); Pan-CK, a glandular cell marker (red); DAPI staining for nuclei (blue). Scale bars = 50 μm. Original magnification: ×400. **f** Percentages of cells in G1, S and G2/M phases in ESCs and Ishikawa with (LV-In-210) or without (LV-In-CN) miR-210-3p knockdown cultured under normoxia or hypoxia conditions. The bars show the mean percentages of three independent experiments ± standard deviation (*n* = 5 for ESCs). *P < 0.05 vs. LV-In-210 under normoxia, ^#^*P* < 0.05 vs. LV-In-CN under hypoxia, one-way ANOVA with LSD for multiple comparisons. **g** Western blot of cell cycle activators Cdc2 and cyclin B1 and inhibitors p53 and p21 in ESCs (left panel) and Ishikawa cells (right panel) infected with LV-In-CN or LV-In-210 under normoxia or hypoxia (*n* = 5 for ESCs)

To identify crucial hypoxia-associated miRNAs, we examined miRNA expressions in ESCs cultured in normoxia and hypoxia using high-throughput miRNA sequencing analysis. Sixteen miRNAs were upregulated more than two-fold and 52 miRNAs were downregulated in hypoxia-cultured ESCs compared with normoxia-cultured ESCs (Supplementary Table 4). MiR-210-3p expression was obviously upregulated after hypoxic treatment for 48 h (Fig. 2a), and qRT-PCR results also confirmed that it was markedly elevated in ESCs and Ishikawa cells cultured in hypoxia compared with normoxic conditions, which was consistent with HIF-1α expression trend of change (Fig. 2b, *P* < 0.05, paired Student's *t-*test, Supplementary Figure 3). Therefore, we selected this miRNA for further analyses.

### MiR-210-3p is overexpressed in endometriotic lesions

MiR-210-3p has been reported to participate in endometriosis development^28,37^, but the mechanism is unclear. We performed qRT-PCR analysis to verify miR-210-3p expression in 15 normal endometria and 15 paired eutopic and ectopic tissues. MiR-210-3p was highly expressed in ectopic lesions and eutopic endometria compared with normal endometria, with the highest level in ectopic lesions (Fig. 2c, *P* < 0.001, Mann–Whitney *U*). We next conducted FISH to visualise miR-210-3p expression in five normal endometria and five paired eutopic endometria and ectopic lesions. MiR-210-3p signal was much stronger in ectopic lesions than in eutopic endometria, with the weakest signal observed in normal endometria (Fig. 2d). Immunofluorescence double staining of CD10 and Pan-cytokeratin revealed strong staining of miR-210-3p both in stroma and gland (Fig. 2e), indicating that miR-210-3p was located in both ESCs and EECs in endometriotic lesions.

### MiR-210-3p knockdown results in G2/M cell cycle arrest under hypoxia

MiR-210 is a robust factor that drives the adaptive response to insufficient oxygen. It also functions in cell cycle regulation and DNA damage response in various diseases^38^, but these functions in endometriosis is unclear. Flow cytometric analysis and cell proliferation were conducted in ESCs and Ishikawa cells infected with LV-In-210 or the negative control LV-In-CN and cultured under normoxia or hypoxia. No change of cell cycle distribution and cell proliferation was observed in LV-In-210-treated cells compared with LV-In-CN-treated cells under normoxia (Fig. 2f, Supplementary Figure 4). However, under hypoxia, the percentage of cells in G2/M phase was significantly increased in LV-In-210-treated cells compared with controls (*P* < 0.05, paired Student *t-*test), while cell proliferation was significantly inhibited (*P* < 0.05, one-way ANOVA). These results demonstrated that knockdown of miR-210-3p under hypoxia resulted in G2/M phase arrest, with no effects in normoxia, implying that miR-210-3p might function in cell cycle regulation in response to hypoxia-associated DNA damage.

### MiR-210-3p knockdown under hypoxia causes cell cycle arrest signal activation

We next examined cyclin B1 and Cdc2, activators of the G2/M transition^39^, and p53 and p21, DNA damage-responsive cell cycle inhibitors^40^. Western blot and qRT-PCR demonstrated that hypoxia significantly increased cyclin B1 and Cdc2 expressions but decreased p53 and p21 expressions in ESCs and Ishikawa cells compared with cells cultured in normoxia (Fig. 2g, Supplementary Figure 5, *P* < 0.05, paired Student *t-*test). However, in cells with miR-210-3p knockdown under hypoxia, both the increase in cyclin B1 and Cdc2 expression and decrease of p53 and p21 levels were compromised compared with LV-In-CN treatment under hypoxia (Fig. 2g, Supplementary Figure 5). Importantly, miR-210-3p knockdown under normoxia had no influence on these molecules expression levels, which was consistent with our flow cytometry results. Given that hypoxia causes DNA damage to ESCs and Ishikawa cells, these results indicated that in response to DNA damage, cells might attenuate cell cycle arrest signal by upregulating miR-210-3p, which in turn maintains cell growth under hypoxic and ROS environment.

### MiR-210-3p decreases BARD1 expression by directly targeting its 3′-UTR

To explore the cellular mechanism of miR-210-3p, we investigated potential targets of miR-210-3p. We compared the transcript patterns of hEM15A cells infected with LV-210 or NC using high-throughput sequencing analysis. The results showed that 27 transcripts were downregulated and 4 transcripts were upregulated in miR-210-3p-overexpressing cells compared with controls (Supplementary Figure 6A, Supplementary Table 5). Moreover, the Gene Ontology (GO) term enrichment analysis showed that many differentially expressed genes participated in cell cycle regulation processes, such as mitosis, chromosome localisation, cell division, microtubule-based movement and spindle and kinetochore microtubule assembly (Supplementary Figure 6B). Three differentially expressed genes attracted our attention, *HMMR*, *BARD1* and *BRIP1*, as these genes are not only involved in cell cycle regulation but the encoded proteins are components of the BRCA1 complex^41^. The BRCA1 complex functions in the DNA damage response and activation of cell cycle checkpoints by quick recruitment of BRCA1 to DNA damage sites andinducing degradation of cell cycle regulatory proteins^42–44^.

**Fig. 3:**
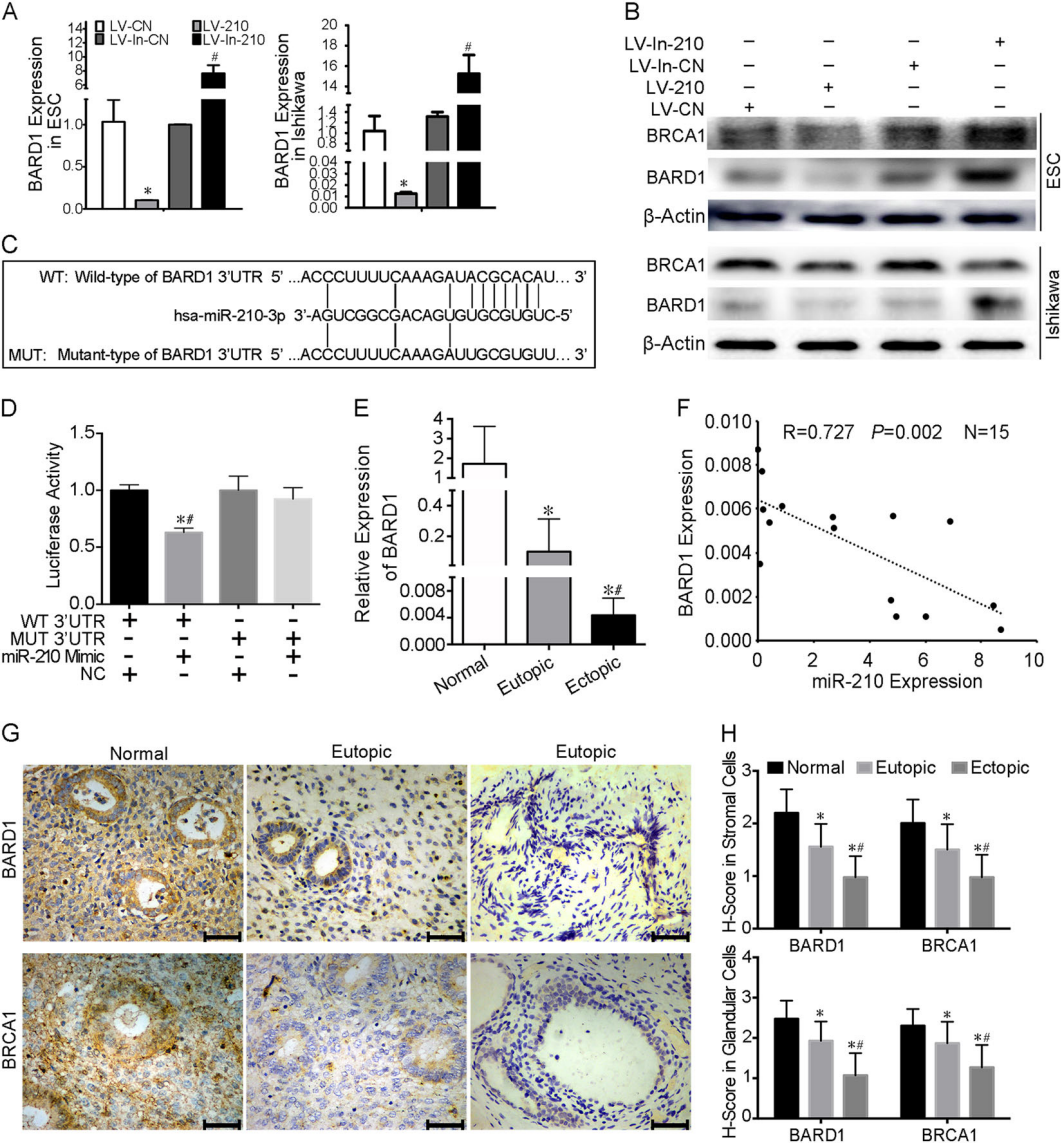
BARD1 is a direct target of miR-210-3p and downregulated in ectopic lesions. **a** qRT-PCR assay of BARD1 mRNA expression in endometrial stromal cells (ESCs) (left panel, *n* = 5) and Ishikawa cells (right panel) infected with LV-CN, LV-210, LV-In-CN or LV-In-210 under normoxia. The bars show the mean value of three independent experiments ± standard deviation. *P < 0.05, LV-210 versus LV-CN; ^#^*P* < 0.05, LV-In-210 versus LV-In-CN; paired Studentʼs *t-*test. **b** Western blot of BARD1 and BRCA1 protein levels in ESCs (*n* = 5) and Ishikawa cells infected with LV-CN, LV-210, LV-In-CN or LV-In-210 under normoxia. β-Actin served as the internal control. **c** Diagram depicting the putative miR-210-3p-binding site within the 3′-UTR of BARD1 and the mutant 3′-UTR of BARD1. **d** 293 T cells were transiently transfected with a luciferase reporter containing wild type (WT) or mutant (MUT) BARD1 3′-UTR, along with miR-210-3p mimic (miR-210 Mimic) or a negative control (NC) (*n* = 3). *P < 0.05 versus treatment of NC with WT 3′-UTR; ^#^*P* < 0.05 versus treatment of miR-210 mimic with MUT 3′-UTR; one-way ANOVA with LSD for multiple comparisons. **e** qRT-PCR assay of BARD1 mRNA expression in normal endometrium and paired eutopic and ectopic tissues (*n* = 15). *P < 0.05 versus normal endometria; ^#^*P* < 0.05 versus eutopic endometria; Mann–Whitney *U* test. **f** Scatter diagram showing linear regression and significant Pearson correlation of BARD1 with miR-210-3p in ectopic lesions based on qRT-PCR results (*n* = 15). *P* < 0.05. **g** Immunohistochemistry photomicrographs of BARD1 and BRCA1 in normal endometrium and paired eutopic and ectopic tissues. Scale bars = 50 µm. Original magnification: ×400. **h** Expressions of BARD1 and BRCA1 in ESCs and endometrial glandular cells by *H*-score (*y*-axis) were significantly higher in normal endometria than in eutopic endometria and ectopic lesions, with ectopic lesions showing the lowest staining (*n* = 27 for normal endometria, *n* = 57 for eutopic endometria, *n* = 57 for ectopic lesions). *P < 0.05 versus normal endometria, ^#^*P* < 0.05 versus eutopic endometria, one-way ANOVA with LSD for multiple comparisons

ESCs and Ishikawa cells infected with LV-210 showed reduced HMMR, BARD1 and BRIP1 mRNA levels, while cells infected with LV-In-210 showed increased HMMR, BARD1 and BRIP1 mRNA levels (Fig. 3a, Supplementary Figure 7A, B). However, only BARD1 protein was changed after LV-210 or LV-In-210 infection (Fig. 3b, Supplementary Figure 7C). Further, BRCA1 protein was decreased after LV-210 infection and increased after LV-In-210 infection (Fig. 3b).

Interestingly, three miRNA target-prediction software (Target Scan, RNA-hybrid and miRanda) predicted BARD1 as the most likely target of miR-210-3p (Fig. 3c). The luciferase activity of the reporter containing the BARD1 3′-UTR with the wild-type seed sequence for miR-210-3p binding was significantly suppressed by co-transfection of miR-210-3p mimic, whereas the reporter with the mutated seed sequence was not affected (Fig. 3d, *P* < 0.05, one-way ANOVA). Together, these results indicated that BARD1 is a direct target of miR-210-3p.

### BARD1 is an indicator of endometriosis severity

By qRT-PCR, we found BARD1 mRNA levels were lower in ectopic and eutopic tissues than in normal endometria, with the lowest expression in ectopic lesions (Fig. 3e, *P* < 0.05, Mann–Whitney *U* test). The correlation between miR-210-3p levels and BARD1 levels in ectopic lesions was identified as negative by linear regression analysis with *R* = 0.727 (Fig. 3f, *P* < 0.05). Immunohistochemistry and *H*-scores revealed that BARD1 was moderately expressed in ESCs and EECs of normal endometria, while BARD1 expression was significantly lower in eutopic and ectopic stroma and gland, with the lowest expressions in stromal cells and glandular cells of ectopic lesions; BRCA1 showed similar expression tendencies (Fig. 3g, h, *P* < 0.05, one-way ANOVA).

Studying the correlation between BARD1 and endometriosis severity may help elucidate the function of miR-210-3p in endometriosis. By chi square tests, we found that the lesion size, chronic pelvic pain score, endometriosis stage and rAFS score were significantly lower in BARD1-positive patients than in BARD1-negative patients (Table 1, *P* < 0.05). Univariate linear regression analyses revealed that lesion size, chronic pelvic pain score, dysmenorrhoea pain score and endometriosis stage were positively correlated with endometriosis severity, which was based on rAFS score, while BARD1 *H*-score showed a negative correlation (Table 2, *P* < 0.01). On multivariate analysis, BARD1 expression still significantly negatively correlated with endometriosis severity (*P* = 0.047), while endometriosis stage showed a positive correlation. These data suggested that BARD1 expression in ectopic lesions is a valuable parameter for indicating endometriosis severity.

**Table 1:** Descriptive characteristics of endometriotic patients with BARD1 expression

	BARD1 negative	BARD1 positive	*P* ^a^
Patients (*n*)	38	19	
Age (years)	30.13 ± 3.45	29.95 ± 3.582	0.615
Height (m)	1.62 ± 0.05	1.59 ± 0.04	0.048
Weight (kg)	54.21 ± 9.11	52.29 ± 6.39	0.415
BMI (kg/m^2^)	20.58 ± 3.26	20.58 ± 2.59	0.999
Haemoglobin (g/dL)	11.52 ± 0.62	11.05 ± 0.21	0.333
Menstrual phage (%)			
Proliferative	33	16	1.000
Luteal	5	3	
EM Endometriosis stage			
I–II	25	19	**0.030** ^b^
III–IV	13	0	
Endometrial thickness	0.725 ± 0.20	0.75 ± 0.27	0.741
rAFS revised American Fertility Society Score	16.45 ± 15.52	4.26 ± + 2.23	**0.001** ^b^
Lesion size (cm)	7.45 ± 10.84	1.32 ± 1.67	**0.021** ^b^
Dysmenorrhoea pain scores	3.79 ± 3.00	2.89 ± 2.54	0.271
Chronic pelvic pain scores	6.47 ± 2.56	4.68 ± 2.14	**0.014** ^b^
AMH Anti-Mullerian Hormone (ng/mL)	2.97 ± 2.14	2.98 ± 2.72	0.889^c^
CA-125 Cancer Antigen 125 (U/mL)	81.10 ± 50.35	65.10 ± 30.74	1.000^c^

*BMI* body mass index

^a^Data was compared by Student *t*-test or by rank test with Mann–Whitney *U* when accorded with normal distribution and homogeneity of variance, or analysed by chi square test when was categorical variables

^b^Values in bold indicated significant correlation

^c^Only a part of the data was evaluable, due to no measurement of the hormones for some patients

**Table 2:** Results of linear regression analyses assessing the relationship between clinical characteristics of endometriosis and the severity of endometriosis by ASRM endometriosis score

Variable	Univariate			Multivariate		
	*B* (SE)	*β*	*P*	*B* (SE)	*β*	*P*
BARD1 expression	−2.568 (0.002)	0.077	**0.002** ^a^	−4.365 (2.197)	0.013	**0.047** ^a^
EM stage	4.237 (1.068)	69.18	**<** **0.001** ^a^	4.489 (2.044)	89.061	**0.028** ^a^
Lesion size	1.999 (0.511)	7.382	**<** **0.001** ^**a**^	0.626 (1.240)	1.869	0.614
Dysmenorrhoea pain scores	0.326 (0.099)	1.385	**0.001** ^**a**^	−0.552 (0.355)	0.576	0.12
Chronic pelvic pain scores	0.81 (0.178)	2.247	**<** **0.001** ^**a**^	1.613 (0.987)	5.02	0.102
Age	0.111 (0.066)	1.118	0.093			
BMI	−0.061 (0.082)	0.941	0.461			

*SE* standard error, *BMI* body mass index

^a^Values in bold indicated significant correlation

### Reduced BARD1 and BRCA1 expressions by hypoxia were dependent on miR-210-3p upregulation

**Fig. 4:**
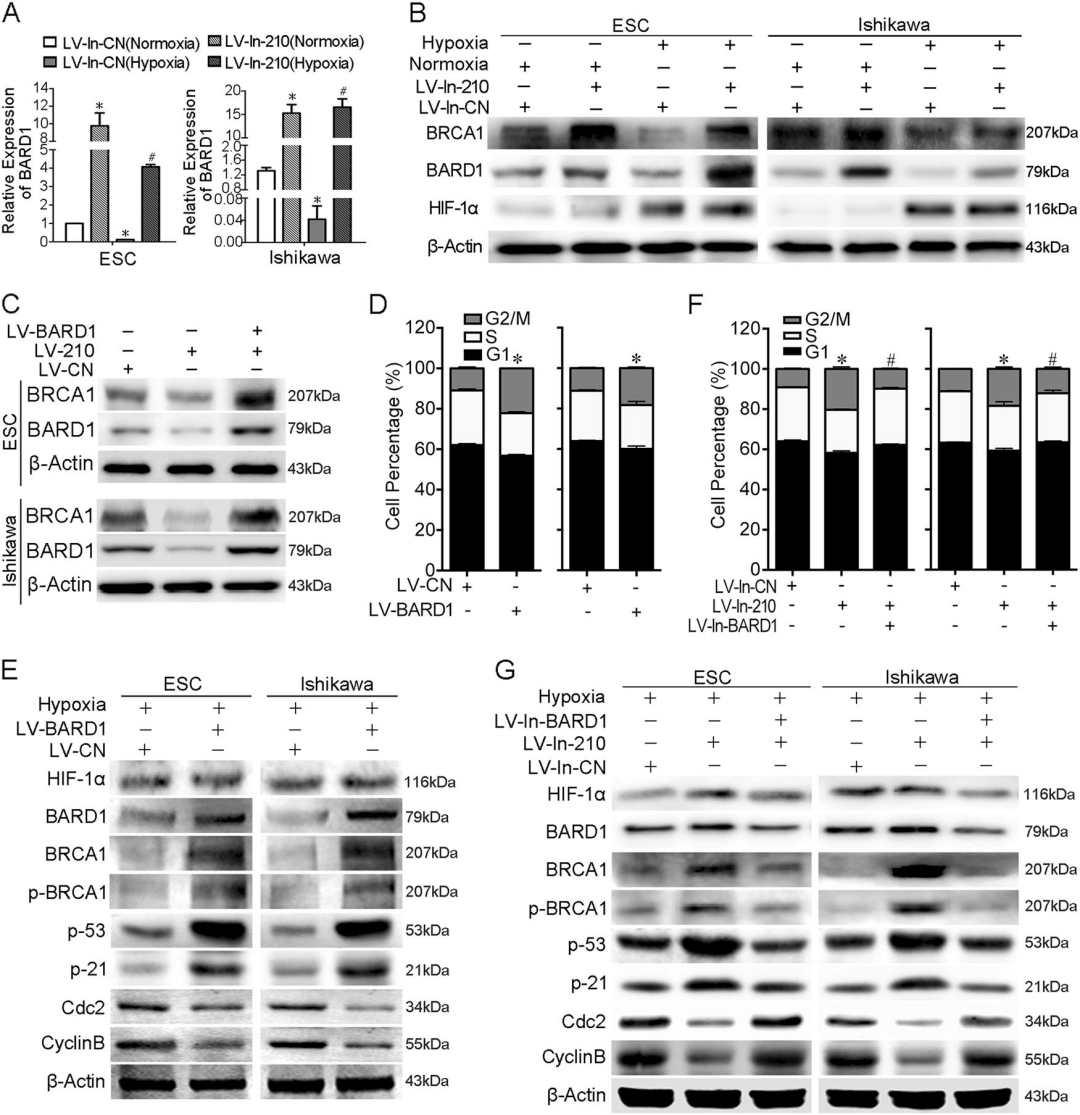
Hypoxia-induced miR-210-3p inhibits BARD1/BRCA1 signalling to avoid cell cycle arrest in hypoxia. **a** qRT-PCR assay of BARD1 mRNA expression in endometrial stromal cells (ESCs) (left panel) and Ishikawa cells (right panel) infected with LV-In-CN or LV-In-210 under normoxia or hypoxia. The bars show the mean value of three independent experiments ± standard deviation. *P < 0.05 versus LV-In-CN (Normoxia); ^#^*P* < 0.05 versus LV-In-CN (Hypoxia) (*n* = 5 for ESCs, paired Studentʼs *t*-test). **b** Western blot of BARD1, BRCA1 and HIF-1α protein levels in ESCs (left panel) and Ishikawa cells (right panel) infected with LV-In-CN or LV-In-210 under normoxia or hypoxia (*n* = 5 for ESCs). β-Actin served as the internal control. **c** Western blot assay of BARD1 and BRCA1 protein levels in ESCs (upper panel) and Ishikawa cells (lower panel) infected with LV-CN, LV-210 or LV-BARD1 under normoxia. **d** Cell cycle distribution of LV-BARD1 or negative control (LV-CN) treated ESCs (left panel, *n* = 5) or Ishikawa cells (right panel) under hypoxia. Each value represents the mean ± standard deviation of three independent experiments. **d** Western blot of BARD1, BRCA1, p-BRCA1, p53, p21, Cdc2, cyclin B1 and HIF-1α protein levels in ESCs (left panel, *n* = 5) and Ishikawa cells (right panel) infected with LV-BARD1 or its negative control (LV-CN) under hypoxia. **e** Cell cycle distribution of LV-In-CN, LV-In-210 or LV-In-210 plus LV-In-BARD1 treated ESCs (upper panel, *n* = 5) or Ishikawa cells (lower panel) under hypoxia. Each value represents the mean ± standard deviation of three independent experiments. **f** Western blot of BARD1, BRCA1, p-BRCA1, p53, p21, Cdc2, cyclin B1 and HIF-1α protein levels in ESCs and Ishikawa cells infected with LV-In-CN, LV-In-210 or LV-In-210 plus LV-In-BARD1 under hypoxia

QRT-PCR and western blot results in both ESCs and Ishikawa cells confirmed that hypoxia decreased BARD1 expression and knockdown of miR-210-3p increased BARD1 expression in hypoxic conditions (Fig. 4a, b). Similarly, the decreased BRCA1 expression under hypoxia conditions was also reversed by miR-210-3p knockdown. These results demonstrated that suppression of BARD1 and BRCA1 expressions under hypoxia was mediated by miR-210-3p upregulation, which might be the cell cycle regulation mechanism of endometrial cells under hypoxic conditions.

### MiR-210-3p inhibits BRCA1 expression via targeting BARD1

BRCA1 functions as both an upstream signal processor and downstream effector in DNA damage responses^45^. We found BRCA1 proteins were decreased after LV-210 infection compared with LV-CN infection, while BRCA1 proteins were increased after LV-210 and LV-BARD1 co-infection compared with LV-210 infection (Fig. 4c; Supplementary Figure 8, *P* < 0.05, paired Student's *t-*test), demonstrating that miR-210-3p suppresses BRCA1 expression by targeting BARD1.

### Complementing BARD1 expression in hypoxia recovers cell cycle arrest activation

Considering the key functions of BARD1 in BRCA1 complex formation and cell cycle regulation response to DNA damage, we speculated that reinforced BARD1 expression in hypoxia might recover activation of the cell cycle checkpoint. LV-BARD1-infected ESCs and Ishikawa cells under hypoxia showed a significantly greater percentage of cells in the G2/M phase and reduced cell proliferation than LV-CN-infected cells (Fig. 4d, *P* < 0.05, Studentʼs *t-*test for cell cycle data and one-way ANOVA for cell proliferation data; Supplementary Figure 9A and B). Moreover, accompanied by BARD1 overexpression, total BRCA1, phosphorylated (activated) BRCA1, p53 and p21 were all upregulated in LV-BARD1-infected ESCs and Ishikawa cells under hypoxia (Fig. 4e). In contrast, the G2/M regulators cyclin B1 and Cdc2 were downregulated (Fig. 4e). These results support the crucial role of BARD1 in cell cycle regulation under hypoxia.

### MiR-210-3p targeting BARD1 maintained cell cycle progression in hypoxia

As our results showed that BARD1 was associated with hypoxia and ROS in endometriosis, we examined the regulatory role of miR-210-3p in targeting BARD1 in the cell cycle and cell proliferation by co-infection with LV-In-210 and LV-In-BARD1. In hypoxia, the increased percentage of G2/M phase cells and suppressed cell growth rate upon miR-210-3p knockdown were both reversed to control levels when miR-210-3p and BARD1 were both knocked down (Fig. 4f, *P* < 0.05, Student *t-*test for data of cell cycle; one-way ANOVA for data of cell growth; Supplementary Figure 9C and D). Western blot also showed that knockdown of both miR-210-3p and BARD1 in hypoxia could attenuate the increases of BRCA1, phosphor-BRCA1, p53 and p21 and reverse the decrease of cyclin B1 and Cdc2, compared with knocking down only miR-210-3p (Fig. 4g). These data demonstrated that hypoxia maintains cell cycle progression by upregulating miR-210-3p, which inhibits the BARD1/BRCA1-mediated cell cycle regulation function in endometriotic cells.

### MiR-210-3p promotes endometriosis progression in vivo

**Fig. 5:**
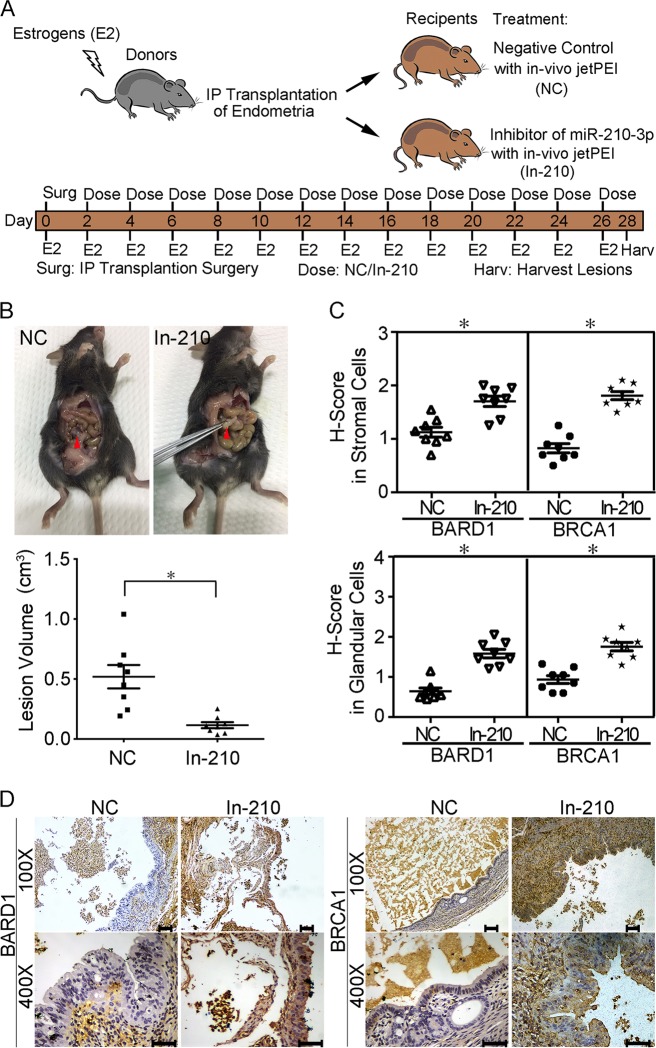
Knockdown of miR-210-3p suppresses endometriosis progression in vivo. **a** Schematic diagram of mouse model of endometriosis. Mice received intraperitoneal (IP) transplantation of uterine tissue from donor mice and were treated with miR-210-3p inhibitor (In-210) or a negative control (NC) using an in vivo-jet PEI delivery agent via intraperitoneal injection every other day. All mice were supplied with 200 mg/kg 17β-estradiol (E2) every other day. After 4 weeks, endometriotic cysts were excised to harvest the extent of endometriosis. **b** Upper panel showing representative visible lesions within the peritoneal cavity of an NC mouse and In-210 mouse. Lower panel showing scatter plot of lesion volumes from NC and In-210 mice (*n* = 8). *P < 0.05 vs. NC, Student's *t-*test. **c** Scatter plot showing expression of BARD1 and BRCA1 in stromal cells and glandular cells from NC or In-210 treated lesions by *H*-score (*y*-axis) (*n* = 8). *P < 0.05 vs. NC, paired Student *t-*test. **d** Immunohistochemistry photomicrographs of BARD1 and BRCA1 expression in NC- or In-210 treated lesions. Scale bar = 100 μm for ×100images. Scale bar = 50 μm for ×400 images

We next explored whether miR-210-3p affects endometriosis development in vivo. Endometriosis model mice were established by intraperitoneal implantation of mouse endometrial fragments, and treated with miR-210-3p inhibitor (In-210) or miRNA NC subsequently (Fig. 5a). Four weeks after implantation, classical endometriosis-like lesions were formed in the pelvic cavity, mesentery or peritoneum (Fig. 5b). The lesion sizes were significantly smaller in In-210 mice than in NC animals (Fig. 5b. *P* < 0.05, Studentʼs *t-*test). Immunohistochemistry showed that ESCs and EECs of endometriotic tissues from NC mice were positive for BARD1 and BRCA1, while In-210 treatment increased BARD1 and BRCA1 expressions in ESCs and EECs (Fig. 5c, d). These data suggest a role for miR-210-3p in promoting lesion formation in vivo.

### Vitamin C alleviates endometriosis progression in vivo

**Fig. 6:**
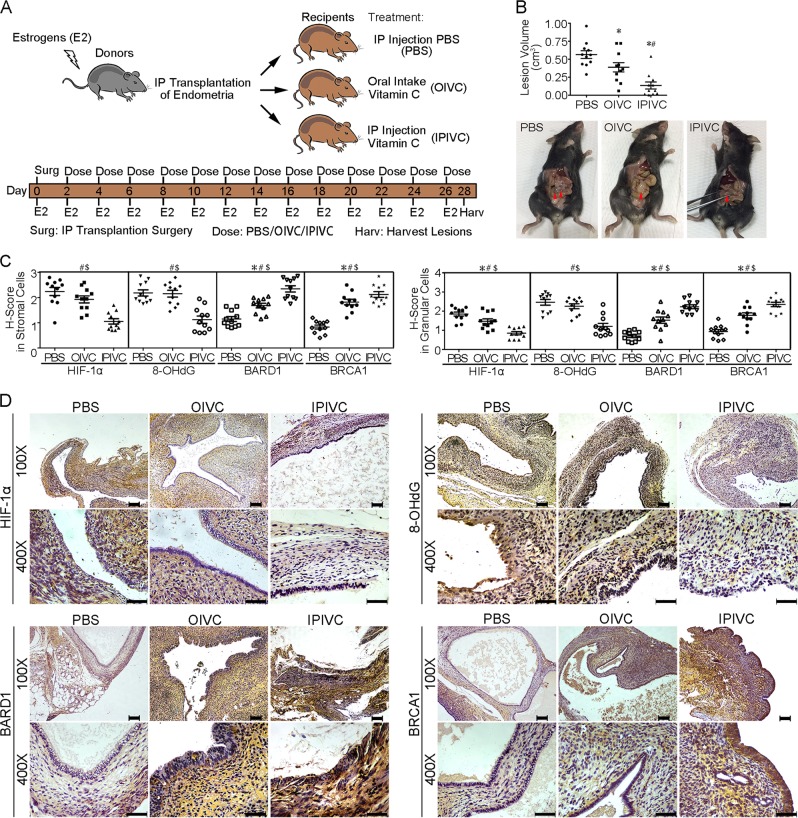
Antioxidant vitamin C suppresses endometriosis progression in vivo by inducing BARD1 and BRCA1 expression. **a** Schematic diagram of mouse model of endometriosis. Mice received intraperitoneal (IP) transplantation of uterine tissue from syngeneic donor mice and were then treated with phosphate-buffered saline (PBS) or vitamin C via intraperitoneal injection (IPIVC) every other day or administered oral intake vitamin C solution (OIVC) every day. All mice were supplied with 200 mg/kg 17β-estradiol every other day. After 4 weeks, endometriotic cysts were excised to harvest the extent of endometriosis. **b** Upper panel shows scatter plot of lesion volumes from PBS, OIVC and IPIVC mice. Lesion sizes were significantly smaller in IPIVC mice than in PBS and OIVC mice, with the smallest cyst volume in IPIVC mice (*n* = 11). *P < 0.05 versus PBS, ^#^*P* < 0.05 versus OIVC, one-way ANOVA with LSD for multiple comparisons. Lower panel shows representative visible lesions within the peritoneal cavity of PBS mouse, OIVC mouse and IPIVC mouse. **c** Expression of HIF-1α, 8-OHdG, BARD1 and BRCA1 in stromal cells (left panel) and glandular cells (right panel) from lesions in PBS, OIVC or IPIVC mice by *H*-score (*y*-axis) (*n* = 11). *P < 0.05, PBS versus OIVC; ^#^*P* < 0.05, PBS versus IPIVC; ^$^*P* < 0.05, OIVC versus IPIVC; one-way ANOVA with LSD for multiple comparisons; the expression of BARD1 and BRCA1 increased in stromal cells and glandular cells of OIVC and IPIVC mice compared with PBS mice (*n* = 11, *P < 0.05, PBS versus OIVC, ^#^*P* < 0.05, PBS versus IPIVC, ^$^*P* < 0.05, OIVC versus IPIVC, one-way ANOVA with LSD for multiple comparisons). **d** Immunohistochemistry photomicrographs of HIF-1α, 8-OHdG, BARD1 and BRCA1 expressions in lesions from PBS, OIVC or IPIVC mice by *H*-score (*y*-axis). Scale bar = 100 μm for ×100 images. Scale bar = 50 μm for ×400 images

Vitamin C is a potent water-soluble chain-breaking antioxidant that effectively prevents OS and DNA damage^46,47^. We next examined the effect of vitamin C on endometriosis. Endometriotic mice were orally administered vitamin C solution or received intraperitoneal vitamin C or PBS injections (Fig. 6a). All mice formed classical endometriosis-like lesions; however, lesion sizes were significantly smaller in vitamin C-treated mice than in PBS-injected mice, with the smallest average volume of cyst in vitamin C-injected mice (Fig. 6b, *P* < 0.05, one-way ANOVA). Moreover, HIF-1α and 8-OHdG expressions in ESCs and EECs of vitamin C-injected mice were dramatically reduced compared with those of PBS-injected mice, with BARD1 and BRCA1 expressions increased (Fig. 6c, d). Oral administration of vitamin C showed similar effects. These results indicate that vitamin C may protect endometriotic cells from OS and DNA damage via reducing HIF-1α and 8-OHdG expression and increasing BARD1 and BRCA1 expression to ultimately alleviate endometriosis progression.

## Discussion

OS and hypoxia are both involved in endometriosis but exhibit inconsistent functions. Excessive OS in endometriotic lesions triggers the DNA damage response and results in cell cycle arrest, while hypoxia is essential for establishment of ectopic lesions. Investigating the mechanisms underlying OS and hypoxia could help clarify their precise contributions to endometriosis progression and reveal potential therapeutic strategies for the disease. Our study illuminates a novel mechanism that resolved OS-generated DNA damage and hypoxia-stimulated cell cycle progression in endometriosis through the activity of a hypoxia-responsive miRNA. We propose upregulated miR-210-3p directly targets BARD1, leading to physiological and biochemical function impairment of the BRCA1 complex, which results in an inactivation of cell cycle arrest in response to DNA damage. Our research also enriches the evidence for using vitamin C as an adjuvant therapy for endometriosis. Although intraperitoneal administration of vitamin C had a suppressive effect on endometriosis development in rat model^34^, the exact mechanism has not been illuminated. Our results found that vitamin C supplementation by oral route or injection decreased the volume of endometriotic cysts and reduced ectopic oxidant expressions, as well as restoring DNA damage responder levels (BARD1 and BRCA1). Vitamin C acts as antioxidant to maintain oxidative balance and may be a promising candidate for endometriosis adjuvant therapy.

Previous studies have suggested that miR-210-3p is involved in endometriotic cell proliferation and participates in the DNA damage response and cell cycle checkpoint in cancer cells via targeting AIFM3 and FGFRL1 (refs. ^28,48^), but the specific molecular mechanisms have not been determined. Here we find that the DNA damage response protein BARD1 is a target of miR-210-3p, and the BRCA1 complex is implicated in cell cycle regulation response to DNA damage under hypoxia in endometriosis. Although previous studies showed that the oxidative imbalanced microenvironment in endometriotic lesions is capable of inducing intracellular kinase-dependent cell growth, especially by PI3K/AKT/mTOR signalling^9^, no study has examined the precise pathway between OS damage and cell cycle in endometriosis. Moreover, no studies have demonstrated roles for BARD1 and BRCA in endometriosis. Their altered expressions in endometriotic lesions imply critical functions for these factors in oxidative imbalance and endometriosis development.

The BARD1-BRCA1 heterodimer participates in DNA double-strand break repair, cell cycle regulation, genomic stability, centrosome duplication and mitotic spindle formation through its E3 ubiquitin ligase activity^49,50^. DNA damage induces BRCA1 phosphorylation^51^, E3 ubiquitin ligase activation and cyclin B1 ubiquitination, which limits the G2/M transition and prevents unscheduled mitotic entry. Our results are consistent with existing evidence, as hypoxia-mediated upregulation of miR-210-3p decreased BARD1 expression and caused BRCA1 protein decrease, which ultimately impaired normal cell cycle regulation (Supplementary Figure 10).

In conclusion, our results show that increased levels of the hypoxia-associated miR-210-3p in endometriotic lesions contribute to endometriosis progression. More studies are needed to explore other critical molecules that may be involved in the endometriotic microenvironment. As a novel target of endometriosis, BARD1 requires more attention to clarify its role in endometriosis progression, prediction and therapy.

## Supplementary information

Supplementary Information

Supplementary Table 1

Supplementary Table 2

Supplementary Table 3

Supplementary Table 4

Supplementary Table 5

Supplementary Figure 1

Supplementary Figure 2

Supplementary Figure 3

Supplementary Figure 4

Supplementary Figure 5

Supplementary Figure 6

Supplementary Figure 7

Supplementary Figure 8

Supplementary Figure 9
