# Supplementary material for: MiR-210-3p protects endometriotic cells from oxidative stress-induced cell cycle arrest by targeting BARD1
Source: Cell Death Dis. 2019 Feb 13;10(2):144. doi: 10.1038/s41419-019-1395-6 (PMC6374490; doi:10.1038/s41419-019-1395-6)
Supplement: Supplementary file 1 — Supplementary Information [file 41419_2019_1395_MOESM1_ESM.doc]

**Supplementary Information**

**Supplementary Methods**

***Inclusion criteria for patients***

The patients recruited in our study were all females 20–35 years of age who underwent laparoscopy or hysteroscopy for endometriosis treatment or to exclude any other gynaecologic-associated disease. Only histologically confirmed middle proliferative endometrial fragments were selected for extraction of ESCs and immunohistochemistry. None of the women had received hormonal treatments and intrauterine contraception for at least 6 months prior to surgery. All participating patients had regular menstrual cycles (672 hours) with confirmation of their menstrual history. Deep infiltrating endometriosis (DIE) was defined as endometriosis infiltrating the retroperitoneal space by 5 mm or more and it presents predominantly in uterosacral ligaments and rectovaginal septum [^3^](#_ENREF_3). Inclusion criteria, tissue collection, processing and storage were conducted according to the World Endometriosis Research Foundation Endometriosis Phenome and Biobanking Harmonisation Project [^4^](#_ENREF_4).

Endometrium undergoes cyclic growth and the levels of some miRNAs change along with the normal endometrial physiology [^5^](#_ENREF_5). To avoid cellular heterogeneity, we selected middle proliferative eutopic endometrial fragments of DIE patients to extract ESCs [^6^](#_ENREF_6). Five eutopic endometrium tissues from 5 DIE patients were collected for extracting ESCs (n=5), and all of them were used for *in vitro* experiments, including hypoxia treatment, alkaline comet assay, lentivirus infection, flow cytometry assay, cell proliferation and migration assay. Fifteen, five and twenty-seven normal proliferative endometria were used for qRT-PCR, FISH and immunohistochemistry, respectively. Fifteen, five and fifty-seven paired eutopic and ectopic tissues were used for qRT-PCR, FISH and immunohistochemistry, respectively.

***Cell culture***

Ishikawa and hEM15A cells were cultured in RPMI 1640 Medium (Gibco, NY, USA) and EMEM (GENOM Biomedical Technology Co., Ltd., Hangzhou, China) supplemented with 10% FBS (Gibco, Australia), respectively.

***Immunohistochemistry assay***

Samples (including 27 normal human endometria, 57 paired human eutopic endometrium and ectopic endometriotic tissues and all mouse model samples) were fixed in 4% paraformaldehyde followed by embedding in paraffin. Slides were incubated in the appropriate primary antibodies overnight at 4°C. Positive controls were included to confirm antibody specificity according to instructions; negative controls were performed by omitting the primary antibodies and using the respective IgGs. H-score of staining results was calculated using the following equation: H-score = Σ Pi (i + 1), where i = intensity of staining, with a value of 1, 2 or 3 (weak, moderate or strong, respectively), and Pi is the percentage of stained cells in total ESCs or EECs, ranging from 0 to 100%. Images were captured and analysed using an optic microscope (BX40, Olympus Optical Corporation, Tokyo, Japan).

***Isolation, purification and culture of ESCs***

We performed primary cell culture to acquire highly pure ESCs according to previous reports [^7^](#_ENREF_7). Briefly, proliferative phase eutopic endometrial tissues obtained by uterine curettage were washed three times with sterile D-Hanks Balanced Salt Solution (HBSS, Jinuo Biotechnology Company, China), carefully minced into 1–2 mm^3^ fragments and incubated in HBSS containing Type I collagenase (100 U/mL, 17100-017, Gibco) at 37°C for 4 h, followed by digestion with DNase I (15 U/mL, 18047019, Life Technologies, USA) for another 30 min. The digested tissues were then filtered through different pore-sized wire sieves to remove cell aggregates and epithelial cells. Undigested material was collected by centrifugation at 100 g for 5 min and the sediment was subjected to another round of digestion. The isolated ESCs were plated in culture dishes in DMEM/F-12 (GENOM Biomedical Technology Co. LTD, Hangzhou, China) supplemented with 10% FBS and incubated at 37°C in 5% CO_2_. The ESCs attached to the bottom of the dish in 8–12 hours and reached 90% confluence in 48 h; the non-attached epithelial cells were removed by rinsing with sterile saline solution and the first passage was conducted to yield ESCs. ESCs were fusiform but more rounded than fibroblasts under an optical microscope. The ESCs from 4–5 generations were collected, cultured and frozen for the following experiments.

***Immunofluorescent authentication of ESCs***

Primary cultured cells were cultured on coverslips, washed gently with PBS twice and fixed with 4% paraformaldehyde. After permeabilisation with PBS-T (0.1% Triton X-100 in PBS solution) for 10 min at 4°C, cells were blocked with 5% BSA for 30 min at room temperature and then incubated with primary monoclonal antibodies against human CD10 (ab126593, Abcam, USA) and Pan-cytokeratins (ab215838, Abcam) at 4°C overnight. Fluorescent-conjugated secondary antibody solution (1:100; MULTI SCIENCES, Hangzhou, China) was used to visualise the signal. DAPI solution (H-1200, Vector Laboratories, USA) was used to stain cell nuclei.

***Alkaline comet assays***

ESCs (n=5) and Ishikawa cells were pre-incubated under hypoxic conditions for 672 hours and then alkaline comet assays were performed using the standard protocol from Trevingen (Gaithersburg, MD, USA). Briefly, ESCs and Ishikawa cells were harvested and resuspended in PBS and then added to agarose gel to achieve a cell concentration of 1×10^5^ cells/mL. The agarose was pipetted onto the comet slides and stored at 4°C in the dark for 10 min before adding pre-chilled lysis buffer for 45 min. The slides were immersed in freshly prepared alkaline solution for 20 min at room temperature, followed by gel electrophoresis at 1 V/cm for 30 min. The slides were washed with 70% ethanol for 5 min and air-dried before staining in diluted 1000x SYBR Gold solution (S11494, Trevingen) and reading with a fluorescence microscope equipped with OpenComet (http://www.cometbio.org/index.html). ESCs and Ishikawa cells were scored to determine the average percentage of DNA damage. The percentage of DNA in the tail was used as the metric of DNA strand breaks and the median value of tail moment was used for statistical analysis [^8^](#_ENREF_8). We calculated the tail lengths, tail DNA percentage and tail olive moments. The experiments were performed independently in triplicate in 5 primary cultured ESCs and Ishikawa cells.

***RNA isolation and qRT-PCR***

Total RNA was isolated using TRIzol reagent (Invitrogen, Carlsbad, CA, USA) according to the manufacturer’s instructions. RNA concentrations were quantified using Nano-Drop 2000 software (Nanodrop, Wilmington, DE, USA). Complementary DNA was prepared from 1 µg RNA using a reverse transcription kit (Promega, USA). The abundance of mRNA and miRNA was evaluated by qRT-PCR using SYBR (DBI, Germany). For the miRNA qRT-PCR assays, we used the internal reference gene U6 as an internal control, and for the mRNA qRT-PCR assay, we used 18S as the internal control. QRT-PCR reactions were run as follows: 40 cycles of 95°C for 15 s and 60°C for 30 s. Expression values were normalised to the arithmetic average of 18S rRNA and the quantification of mRNA abundance was based on the threshold cycle (Ct) as 2-△(△Ct), where △Ct=Ct(target gene)-Ct(18S) and △(△Ct)=Ct(experimental group)-Ct(control group). To evaluate miR-210-3p levels, expression values were normalised to the arithmetic average of U6 and the quantification of miR-210-3p abundance was based on the threshold cycle (Ct) as 2-△(△Ct), where △Ct=Ct(miR-210-3p)-Ct(U6) and △(△Ct)=Ct(experimental group)-Ct(control group). The experiments were performed independently in triplicate in ESCs (n=5) and Ishikawa cells. The primer sequences are listed in Supplementary Table 1.

***Lentiviral infection***

Lentivirus-infected ESCs and Ishikawa were observed under fluorescence microscopy to confirm infection efficiency after 24 hours of culture. Cell medium was changed every 24 h. QRT-PCR was performed to confirm the efficiency of lentivirus infection. Cells were only used for *in vitro* experiments if the infection efficiency was more than 80%.

***Western blot assay***

Total proteins were extracted from treated cells using radio-immunoprecipitation assay buffer (R0020, Solarbio, China) at 4°C. Protein concentration was determined using a bicinchoninic acid protein quantitative analysis kit (MK164229, Pierce, San Francisco, CA, USA) according to the manufacturer’s instructions. Protein samples were separated by 7.5% SDS-PAGE or 10% SDS-PAGE and transferred to PVDF membranes (Bio-Rad, Hercules, CA, USA). The membranes were incubated in TBS supplemented with 5% BSA at room temperature for 1 hour prior to incubation at 4°C overnight with the primary antibodies. The PVDF membranes were washed three times with TBS before incubation with HRP-labelled secondary antibodies (IRDye 800CW, LI-COR, USA) for 30 min at room temperature. Signals were developed with ECL reagents (Pierce, Thermo Scientific, Rockford, IL, USA) and digitised on an Image Quant LAS 4000 mini (28955813, GE Healthcare Life Sciences, Pittsburgh, PA, USA). Quantification of signals was carried out using Image J software. The experiments were performed independently in triplicate in ESCs (n=5) and Ishikawa cells. The primary antibodies and concentrations used in Western blot assay are listed in Supplementary Table 2.

***Luciferase assays***

Luciferase reporter vectors (pmiR-RB-REPORT) containing the wild-type seed sequence of BARD1 3′-UTR that binds with miR-210-3p or the mutant sequence (as indicated in Figure 3C) were purchased from Ribobio (Guangzhou, China). For luciferase assays, 293T cells were cultured in 96-well plates and transfected with either wild-type or mutant pmiR-RB-REPORT, which contains both firefly and renilla luciferase genes, together with the miR-210-3p mimic or mimic-NC (negative control) (RiboBio). Luciferase activity was evaluated 48 hours post-transfection using the Dual Luciferase Reporter Assay System (Promega, Madison, WI, USA) according to the manufacturer’s instructions.

***Cell cycle assay***

ESCs and Ishikawa cells were starved for 12 hours followed by LV-In-CN or LV-In-210 infection and cultured under normoxia or hypoxia simultaneously for 48 h. In other experiments, ESCs and Ishikawa were starved for 12 hours before infection with LV-BARD1 or the negative control under hypoxia culture. We also performed co-infection experiments, in which cells were infected with LV-In-210 for 24 hours before infection with LV-In-BARD1 for another 24 hours under hypoxia culture. The infected ESCs and Ishikawa cells were harvested, washed twice with cold PBS and fixed with ice-cold 70% ethanol overnight at 4°C. Cells were washed twice with PBS and then incubated with 0.5 ml PBS containing 50 µg/mL propidium iodide, 0.2% Triton X-100 and 100 µg/ml DNase-free RNase (Sigma-Aldrich, St. Louis, MO, USA) at room temperature in the dark for 30 min. The experiments were performed independently in triplicate in ESCs (n=5) and Ishikawa.

***Cell proliferation analysis***

ESCs and Ishikawa cells were infected by indicated lentivirus to enhance or suppress BARD1 expression or interfered miR-210-3p expression 3 days before seeded in a 96-well plate. Cell growth rate was determined using a CCK-8 kit (Tojindo, Shanghai, China) according to the manufacturer’s instructions. Optical density (OD) was measured for 4 days.

***Mouse model of endometriosis***

Eight-week-old female C57BL6 mice (23–25 g) were purchased from Shanghai Animal Centre, Chinese Academy of Science, and housed in our animal centre for 1 week before operation. Mice that showed regular 4-day or 5-day oestrous cycles were used. Surgical endometriosis was induced by transplanting allogeneic uterine tissue onto the intestinal mesentery [^9^](#_ENREF_9). Briefly, the donor mice were pre-treated with 200 µg/kg 17β-estradiol (Sigma-Aldrich, St. Louis, MO, USA) every 24 hours by intraperitoneal injection for 168 hours. The donor uterus was excised from the uterine horn and cervix uteri, two strips were cut longitudinally with micro-scissors and the endometrial mucosa was exposed. Endometrial tissue with an approximate size of 0.5 cm × 0.3 cm was sutured on both ends using 6/0 polypropylene and then stitched to the bowel serosa of the intestinal mesentery. To minimise the effect of the environment, the operation time was limited to 15 min. After surgery, each ovariectomized receptor mouse was administered 17β-estradiol (200 µg/kg, Sigma-Aldrich) by intraperitoneal injection every 48 hours [^10^](#_ENREF_10).

Endometriosis C57BL6 mice were randomised into two groups (n=8 per group): the miRNA negative control (NC) and the miR-210-3p inhibitor administration group (In-210). Mice in the In-210 group were intraperitoneally injected with 16 μl vivo-jet PEI delivery agent (Polyplus, NY, USA) containing 100 μg of miR-210-3p inhibitor in 5% glucose; mice in the NC group were injected with 16 μl vivo-jet PEI delivery agent containing miRNA negative control in 5% glucose.

To examine the effects of vitamin C in endometriosis, thirty-three endometriosis C57BL6 mice were randomised into three groups (n=11 per group): the control group (intraperitoneal injection PBS, abbreviated as PBS), the oral In-Take group (oral In-Take vitamin C, abbreviated as OIVC) and the Injection group (intraperitoneal injection vitamin C, abbreviated as IPIVC). Each mouse in the OIVC group was given 2.5 mg of vitamin C (H20056946, Shandong Pharmaceutical Company Limited, Zibo, China) in 1 mL of purified water via oral gavage every 24 hours [^11^](#_ENREF_11); each mouse in the Injection group was given 500 mg/kg of vitamin C (Redoxon ampoule, Bayer Turkish Chemical Industry Trade Co. Ltd., Istanbul, Turkey) by intraperitoneal injection every 48 hours. Each mouse in the control group was given an equal volume of PBS to replace vitamin C by intraperitoneal injection every 48 hours. We repeated these treatments along with intraperitoneal injection of oestrogen (200 µg/kg) every 48 hours until 4 weeks at the end of the experiments (a schematic diagram is shown in Figure 6A). All mice were executed by luxation to obtain ectopic lesions. The volumes of endometriotic cysts were measured, and the technicians who measured the volume were blinded to the treatment. The implants grew into macroscopic ellipsoidal cysts that contained both endometrial glands and stroma; these implants were used for immunohistochemistry assay performed with monoclonal antibodies described in Supplementary Table 2.

***Supplementary Figure Legends***

**Supplementary Figure 1** Immunofluorescent authentication of ESCs.

ESCs were identified by positive immunofluorescence staining for the CD10 marker of ESCs (left panel). ESCs were negative for Pan-cytokeratin, a marker of endometrial epithelial cells (middle panel). The right panel shows merging of both CD10 and Pan-Cytokeratins signals, with DAPI (blue) staining for cell nuclei.

**Supplementary Figure 2** Hypoxic treatment caused severe DNA damage to ESCs and Ishikawa cells.

Quantatitive analysis of DNA damage based on fluorescence microscope images from alkaline comet assays, showing comet tail length (left panel), percentage of tail DNA (middle panel) and tail olive moment (right panel). (A) ESCs; (B) Ishikawa cells. At lease 50 nuclei were measured for each case. The alkaline comet assay was conducted in five ESCs and showed similar results; the results from one representative experiment are shown. **P* < 0.05, hypoxia vs. normoxia, Student *t* test or Mann-Whitney U.

**Supplementary Figure 3** Hypoxia increased protein expression of HIF-1α in ESCs.

Five ESCs and Ishikawa were pretreated in hypoxic culture conditions for the indicated time. HIF-1α expression level was determined by Western blot. β-Actin was used as an internal control.

**Supplementary Figure 4** Flow cytometric assays for ESCs and Ishikawa cells with or without miR-210-3p knockdown cultured under normoxia or hypoxia conditions.

1. Representative cell cycle results from ESCs (upper panel) and Ishikawa (low panel) with (LV-In-210) or without (LV-In-CN) miR-210-3p knockdown cultured under normoxia or hypoxia conditions.
2. Cell proliferation of ESCs (left panel) and Ishikawa (right panel) with (LV-In-210) or without (LV-In-CN) miR-210-3p knockdown cultured under normoxia or hypoxia conditions. The growth rate of cells was measured as an increase in the absorbance over indicated time. **P* < 0.05, Hypoxia-In-CN vs. Normoxia-In-CN; #*P* < 0.05, Hypoxia-In-210 vs Hypoxia-In-CN; one-way ANOVA.

**Supplementary Figure 5** Knockdown of miR-210-3p under hypoxia caused cell cycle arrest signal activation.

QRT-PCR assay of mRNA levels of cell cycle activators Cdc2 and cyclin B1 and cell cycle inhibitors p53 and p21 in 5 ESCs (upper panel) and Ishikawa cells (lower panel) infected with LV-In-CN or LV-In-210 under normoxia or hypoxia. HIF-1α expression was upregulated regardless of miR-210-3p expression. **P* < 0.05 vs. LV-In-Cn (Normoxia); # versus LV-In-210 (Normoxia); $ versus LV-In-CN (Hypoxia), Student *t* test.

**Supplementary Figure 6** Differentially expressed genes in LV-210 and LV-CN cells.

1. Differentially expressed genes between miR-210-3p overexpressing (LV-210) and control (LV-CN) hEM15A cells were determined by high-throughput sequencing. The abscissa of volcano plot represents the fold-changes of differentially expressed genes between the two groups, and the ordinate represents the statistical significance of the changes.
2. Heat map shows the differentially expressed genes between LV-210 and LV-CN cells. Each column represents one library, and the colour bar indicates relative expression level ranging from high (red) to low (green).
3. Gene ontology enrichment analysis of differentially expressed genes between LV-210 and LV-CN cells, showing biological processes of Gene Ontology terms (green), cellular components of Gene Ontology terms (orange) and molecular functions of Gene Ontology terms (red).

**Supplementary Figure 7** Validation of differentially expressed genes identified by high-throughput sequencing using qRT-PCR and Western blot.

1. QRT-PCR results showing the relative expressions of BRIP1 and HMMR mRNAs in miR-210-3p overexpressing (LV-210) and control (LV-CN) ESCs. **P* < 0.05, LV-210 vs. LV-CN, Student *t* test.
2. QRT-PCR results showing the relative expressions of BRIP1 and HMMR mRNAs in miR-210-3p knockdown (LV-In-210) and control (LV-In-CN) ESCs. **P* < 0.05, LV-In-210 vs. LV-In-CN, Student *t* test.
3. Western blot of BRIP1 and HMMR levels in miR-210-3p overexpressing (LV-210) and control (LV-CN) ESCs and miR-210-3p knockdown (LV-In-210) and control (LV-In-CN) ESCs.

**Supplementary Figure 8** BARD1 expression in LV-210 and LV-BARD1 infected cells.

QRT-PCR of BARD1 mRNA in ESCs (A) and Ishikawa (B) cells infected with LV-CN, LV-210 or LV-210 together with LV-BARD1 under normoxia. **P* < 0.05 versus LV-CN; #*P* < 0.05 versus LV-210 (n=5 for ESCs, paired Student *t* test).

**Supplementary Figure 9** Flow cytometric assays for cell cycle phase distribution in ESCs and Ishikawa cells under hypoxia.

1. Representative cell cycle results from ESCs (upper panel) and Ishikawa cells (lower panel) infected by LV-CN and LV-BARD1.
2. Cell proliferation of ESCs (upper panel) and Ishikawa (lower panel) with (LV-BARD1) or without (LV-CN) BARD1 overexpression cultured under hypoxia condition. The growth rate of cells was measured as an increase in the absorbance over indicated time. **P* < 0.05, one-way ANOVA.
3. Representative cell cycle results from ESCs (upper panel) and Ishikawa cells (lower panel) infected by LV-In-CN, LV-In-210 and LV-In-210 together with LV-In-BARD1.
4. Cell proliferation of ESCs (upper panel) and Ishikawa (lower panel) with treatments of LV-In-CN, LV-In-210 or LV-In-210 plus LV-In-BARD1 cultured under hypoxia condition. The growth rate of cells was measured as an increase in the absorbance over indicated time. **P* < 0.05, LV-In-210 vs LV-In-CN; # *P* < 0.05, In-210 & In-BARD1 vs LV-In-210; one-way ANOVA.

**Supplementary Figure 10** Working model.

Regulation of oxidative-stress-generated DNA damage and hypoxia-stimulated cell cycle progression in endometriosis is mediated by the activity of a hypoxia-responsive miRNA. Upregulated miR-210-3p directly targets BARD1, leading to physiological and biochemical function impairment of the BRCA1 complex, which results in an inactivation of cell cycle arrest.

**Reference:**

1 Koninckx, P. R., Meuleman, C., Demeyere, S., Lesaffre, E. & Cornillie, F. J. Suggestive evidence that pelvic endometriosis is a progressive disease, whereas deeply infiltrating endometriosis is associated with pelvic pain. *Fertil Steril* **55**, 759-765 (1991).

2 Koninckx, P. R., Ussia, A., Adamyan, L., Wattiez, A. & Donnez, J. Deep endometriosis: definition, diagnosis, and treatment. *Fertil Steril* **98**, 564-571 (2012).

3 Kinkel, K., Frei, K. A., Balleyguier, C. & Chapron, C. Diagnosis of endometriosis with imaging: a review. *Eur Radiol* **16**, 285-298 (2006).

4 Fassbender, A. *et al.* World Endometriosis Research Foundation Endometriosis Phenome and Biobanking Harmonisation Project: IV. Tissue collection, processing, and storage in endometriosis research. *Fertil Steril* **102**, 1244-1253 (2014).

5 Kuokkanen, S. *et al.* Genomic profiling of microRNAs and messenger RNAs reveals hormonal regulation in microRNA expression in human endometrium. *Biology of reproduction* **82**, 791-801 (2010).

6 Saare, M. *et al.* Challenges in endometriosis miRNA studies - From tissue heterogeneity to disease specific miRNAs. *Biochimica et biophysica acta* **1863**, 2282-2292 (2017).

7 Lin, X. *et al.* Hypoxia Promotes Ectopic Adhesion Ability of Endometrial Stromal Cells via TGF-beta1/Smad Signaling in Endometriosis. *Endocrinology* **159**, 1630-1641 (2018).

8 Gyori, B. M., Venkatachalam, G., Thiagarajan, P. S., Hsu, D. & Clement, M. V. OpenComet: an automated tool for comet assay image analysis. *Redox Biol* **2**, 457-465 (2014).

9 Pelch, K. E., Sharpe-Timms, K. L. & Nagel, S. C. Mouse model of surgically-induced endometriosis by auto-transplantation of uterine tissue. *J Vis Exp*, e3396 (2012).

10 Li, J., Dai, Y., Zhu, H., Jiang, Y. & Zhang, S. Endometriotic mesenchymal stem cells significantly promote fibrogenesis in ovarian endometrioma through the Wnt/beta-catenin pathway by paracrine production of TGF-beta1 and Wnt1. *Hum Reprod* **31**, 1224-1235 (2016).

11 Durak, Y. *et al.* Effect of vitamin C on the growth of experimentally induced endometriotic cysts. *J Obstet Gynaecol Res* **39**, 1253-1258 (2013).
