# Supplementary material for: MiR-210-3p protects endometriotic cells from oxidative stress-induced cell cycle arrest by targeting BARD1
Source: Cell Death Dis. 2019 Feb 13;10(2):144. doi: 10.1038/s41419-019-1395-6 (PMC6374490; doi:10.1038/s41419-019-1395-6)
Supplement: Supplementary file 2 — Supplementary Table 1 [file 41419_2019_1395_MOESM2_ESM.docx]

**Supplementary Table 1**. Clinical characteristics of control and endometriotic patients.

|  |  | Control | Endometriosis | *P* |
| --- | --- | --- | --- | --- |
| Patients (n) | | 47 | 82 |  |
| Age (years) | | 32.18±5.05 | 30.30±3.83 | ***0.019**** |
| Height (m) | | 1.61±0.04 | 1.61±0.05 | 1.000 |
| Weight (Kg) | | 54.71±6.89 | 53.54±7.64 | 0.388 |
| BMI (Kg/m2) | | 21.13±2.28 | 20.55±2.81 | 0.230 |
| Menstrual phage (%) | |  |  |  |
|  | Proliferative | 46 | 69 | 0.247 |
|  | Luteal | 3 | 11 |  |
| Endometrial Thickness | | 0.704±0.075 | 0.735±0.212 | 0.335 |
| Dysmenorrhea pain scores | | 0.220±1.093 | 3.570±2.889 | ***<0.001**** |
| Chronic pelvic pain scores | | 0.240±1.255 | 5.840±2.750 | ***<0.001**** |
| AMH (ng/mL) | | 1.491±0.800 | 2.897±1.832 | 0.092^#^ |
| CA-125 (U/mL) | | 14.434±4.091 | 58.961±43.720 | ***0.001**** |
| E2 (estradiol, ng/L) | | 46.238±1.625 | 42.74±1.617 | 0.274^#^ |
| Serum Progesterone (ug/L) | | 0.952±0.526 | 2.162±2.924 | 0.358^#^ |
| LH (IU/L) | | 3.424±1.625 | 4.274±1.617 | 0.078^#^ |
| FSH (IU/L) | | 6.667±1.766 | 7.660±3.196 | 0.168^#^ |

* Values in bold indicated significant correlation. Data was compared by Student *t*-test or by rank test with Mann-Whitney U when accorded with normal distribution and homogeneity of variance, or analysed by chi-square test when was categorical variables.

#Only a part of the data was evaluable, due to no measurement of the hormones for some patients.
