# Supplementary material for: MiR-210-3p protects endometriotic cells from oxidative stress-induced cell cycle arrest by targeting BARD1
Source: Cell Death Dis. 2019 Feb 13;10(2):144. doi: 10.1038/s41419-019-1395-6 (PMC6374490; doi:10.1038/s41419-019-1395-6)
Supplement: Supplementary file 3 — Supplementary Table 2 [file 41419_2019_1395_MOESM3_ESM.docx]

**Supplementary Table 2**. Information of antibodies used in western blot and immunohistochemistry.

| Antigen | Catalog number | Dilution in WB | Dilution in IHC | Producer | Country |
| --- | --- | --- | --- | --- | --- |
| HIF-1α | ab16066 | 1:1000 | 1:200 | Abcam | UK |
| BARD1 | sc-11438 | 1:1000 | 1:200 | Santa Cruz Biotechnology | USA |
| BRCA1 | ab191042 | 1:800 | 1:200 | Abcam | UK |
| 8-OHdG | ab10802 | NA | 1:1000 | Abcam | UK |
| p-BRCA1 | ab90528 | 1:1000 | NA | Cell Signaling Technology | USA |
| p53 | 9286 | 1:1000 | NA | Cell Signaling Technology | USA |
| p21 | 2947 | 1:1000 | NA | Cell Signaling Technology | USA |
| Cdc2 | 9116S | 1:1000 | NA | Cell Signaling Technology | USA |
| CyclinB1 | 12231S | 1:1000 | NA | Cell Signaling Technology | USA |
| β-actin | sc-69879 | 1:1000 | NA | Santa Cruz Biotechnology | USA |

NA, not applied.
