# Supplementary material for: MiR-210-3p protects endometriotic cells from oxidative stress-induced cell cycle arrest by targeting BARD1
Source: Cell Death Dis. 2019 Feb 13;10(2):144. doi: 10.1038/s41419-019-1395-6 (PMC6374490; doi:10.1038/s41419-019-1395-6)
Supplement: Supplementary file 4 — Supplementary Table 3 [file 41419_2019_1395_MOESM4_ESM.docx]

**Supplementary Table 3**. List of primers used in qRT-PCR analysis.

| Gene Name | Sequence |
| --- | --- |
| HIF-1α | Forward 5’- TGAGCCTAATAGTCCCAGTGAA-3’  Forward 5’- TAGGGAGCTAACATCTCCAAGT-3’ |
| BARD1 | Forward 5’- GTTGTTCCTGGTGATGCAGTTC-3’  Forward 5’- ATGGTGTTTGAAGGTTCCCCA-3’ |
| HMMR | Forward 5’- TGGCGTCTCCTCTATGAAGAA-3’  Forward 5’- GCTGACAGCGGAGTTTTGAT-3’ |
| BRIP1 | Forward 5’- ACAATGCCCGTGCTGTCATA-3’  Forward 5’- AGCTCCCCAATCATTTCTGTGT-3’ |
| P53 | Forward 5’- TGTGACTTGCACGTACTCCC-3’  Reverse 5’- ACCATCGCTATCTGAGCAGC-3’ |
| P21 | Forward 5’-AAAGGATGACAAGCAGAGAGCC-3’  Reverse 5’-GTGACAGGTCCACATGGTCT-3’ |
| Cdc2 | Forward 5’- CTGTGTTACACCACGCTCCT-3’  Reverse 5’- TCTTCACAAACTCGTCCCCG-3’ |
| CyclinB1 | Forward 5’-GTGTAGGTCCTTGGCTGGTC-3’  Reverse 5’-CCTGCCATGTTGATCTTCGC-3’ |
| 18S | Forward 5’- CTCTTAGCTGAGTGTCCCGC-3’  Reverse 5’- CTGATCGTCTTCGAACCTCC-3’ |
