# Supplementary material for: MiR-210-3p protects endometriotic cells from oxidative stress-induced cell cycle arrest by targeting BARD1
Source: Cell Death Dis. 2019 Feb 13;10(2):144. doi: 10.1038/s41419-019-1395-6 (PMC6374490; doi:10.1038/s41419-019-1395-6)
Supplement: Supplementary file 5 — Supplementary Table 4 [file 41419_2019_1395_MOESM5_ESM.docx]

**Supplementary Table 4**. List of Differentially Expression miRs identified by miR-Sequencing in ESC cultured under hypoxia or normoxia.

| miRNA_ID | ESC1-N | ESC2-N | ESC1-H | ESC2-H | Up/Down | \|log2(foldchange)\| | P-value |
| --- | --- | --- | --- | --- | --- | --- | --- |
| hsa-miR-3177-3p | 0.000 | 0.000 | 1.185 | 0.963 | Up | 13.391 | 0.000 |
| hsa-miR-150-5p | 0.000 | 0.000 | 1.086 | 0.963 | Up | 13.323 | 0.000 |
| hsa-miR-502-5p | 0.000 | 0.000 | 0.987 | 1.060 | Up | 13.321 | 0.000 |
| hsa-miR-320d | 0.000 | 0.000 | 0.000 | 1.252 | Up | 12.612 | 0.010 |
| hsa-miR-541-5p | 0.000 | 0.000 | 0.000 | 1.060 | Up | 12.371 | 0.010 |
| hsa-let-7g-3p | 0.000 | 0.000 | 0.987 | 0.000 | Up | 12.269 | 0.000 |
| hsa-miR-33b-3p | 0.000 | 0.000 | 0.000 | 0.963 | Up | 12.234 | 0.000 |
| hsa-miR-449c-5p | 0.000 | 0.000 | 0.000 | 0.963 | Up | 12.234 | 0.000 |
| hsa-miR-675-5p | 0.000 | 0.000 | 0.000 | 0.963 | Up | 12.234 | 0.000 |
| hsa-miR-4466 | 0.000 | 0.000 | 0.000 | 0.963 | Up | 12.234 | 0.000 |
| hsa-miR-210-5p | 1.573 | 1.294 | 7.109 | 7.322 | Up | 2.332 | 0.000 |
| hsa-miR-4443 | 0.000 | 1.693 | 3.456 | 2.890 | Up | 1.907 | 0.000 |
| hsa-miR-210-3p | 73.816 | 78.058 | 263.509 | 273.881 | Up | 1.823 | 0.000 |
| hsa-miR-6134 | 0.000 | 1.195 | 1.284 | 1.252 | Up | 1.086 | 0.063 |
| hsa-miR-34a-3p | 1.081 | 0.000 | 1.185 | 1.060 | Up | 1.054 | 0.097 |
| hsa-miR-3200-3p | 1.180 | 0.000 | 1.481 | 0.963 | Up | 1.051 | 0.097 |
| hsa-miR-548a-3p | 1.278 | 1.991 | 0.000 | 0.000 | Down | 13.997 | 0.000 |
| hsa-miR-5699-5p | 1.180 | 1.991 | 0.000 | 0.000 | Down | 13.953 | 0.000 |
| hsa-miR-548u | 1.278 | 1.294 | 0.000 | 0.000 | Down | 13.651 | 0.000 |
| hsa-miR-3687 | 1.474 | 1.095 | 0.000 | 0.000 | Down | 13.649 | 0.000 |
| hsa-miR-1248 | 0.983 | 1.493 | 0.000 | 0.000 | Down | 13.596 | 0.001 |
| hsa-miR-548e-5p | 1.180 | 1.294 | 0.000 | 0.000 | Down | 13.594 | 0.001 |
| hsa-miR-548f-5p | 1.278 | 1.195 | 0.000 | 0.000 | Down | 13.594 | 0.001 |
| hsa-miR-7975 | 1.180 | 0.996 | 0.000 | 0.000 | Down | 13.409 | 0.001 |
| hsa-miR-4467 | 0.983 | 0.996 | 0.000 | 0.000 | Down | 13.272 | 0.002 |
| hsa-miR-3613-5p | 0.000 | 1.493 | 0.000 | 0.000 | Down | 12.866 | 0.007 |
| hsa-miR-579-5p | 1.474 | 0.000 | 0.000 | 0.000 | Down | 12.848 | 0.007 |
| hsa-miR-184 | 0.000 | 1.394 | 0.000 | 0.000 | Down | 12.767 | 0.013 |
| hsa-miR-1227-3p | 0.000 | 1.294 | 0.000 | 0.000 | Down | 12.660 | 0.024 |
| hsa-miR-3928-3p | 0.000 | 1.294 | 0.000 | 0.000 | Down | 12.660 | 0.024 |
| hsa-miR-5001-3p | 1.278 | 0.000 | 0.000 | 0.000 | Down | 12.641 | 0.024 |
| hsa-miR-200a-3p | 0.000 | 1.195 | 0.000 | 0.000 | Down | 12.544 | 0.024 |
| hsa-miR-3605-3p | 1.180 | 0.000 | 0.000 | 0.000 | Down | 12.526 | 0.024 |
| hsa-miR-206 | 0.000 | 1.095 | 0.000 | 0.000 | Down | 12.419 | 0.024 |
| hsa-miR-487b-5p | 0.000 | 1.095 | 0.000 | 0.000 | Down | 12.419 | 0.024 |
| hsa-miR-4685-3p | 0.000 | 1.095 | 0.000 | 0.000 | Down | 12.419 | 0.024 |
| hsa-miR-1245b-3p | 0.000 | 1.095 | 0.000 | 0.000 | Down | 12.419 | 0.024 |
| hsa-miR-196b-3p | 1.081 | 0.000 | 0.000 | 0.000 | Down | 12.400 | 0.024 |
| hsa-miR-134-3p | 0.000 | 0.996 | 0.000 | 0.000 | Down | 12.281 | 0.000 |
| hsa-miR-433-5p | 0.000 | 0.996 | 0.000 | 0.000 | Down | 12.281 | 0.000 |
| hsa-miR-616-5p | 0.000 | 0.996 | 0.000 | 0.000 | Down | 12.281 | 0.000 |
| hsa-miR-1228-3p | 0.000 | 0.996 | 0.000 | 0.000 | Down | 12.281 | 0.000 |
| hsa-miR-7641 | 0.000 | 0.996 | 0.000 | 0.000 | Down | 12.281 | 0.000 |
| hsa-miR-545-3p | 0.983 | 0.000 | 0.000 | 0.000 | Down | 12.263 | 0.000 |
| hsa-miR-887-5p | 0.983 | 0.000 | 0.000 | 0.000 | Down | 12.263 | 0.000 |
| hsa-miR-942-5p | 0.983 | 0.000 | 0.000 | 0.000 | Down | 12.263 | 0.000 |
| hsa-miR-3661 | 0.983 | 0.000 | 0.000 | 0.000 | Down | 12.263 | 0.000 |
| hsa-miR-4531 | 0.983 | 0.000 | 0.000 | 0.000 | Down | 12.263 | 0.000 |
| hsa-miR-664b-3p | 0.983 | 0.000 | 0.000 | 0.000 | Down | 12.263 | 0.000 |
| hsa-miR-7111-3p | 0.983 | 0.000 | 0.000 | 0.000 | Down | 12.263 | 0.000 |
| hsa-miR-188-5p | 2.457 | 3.186 | 0.987 | 0.000 | Down | 2.515 | 0.000 |
| hsa-miR-3074-3p | 1.573 | 2.589 | 0.000 | 0.963 | Down | 2.111 | 0.004 |
| hsa-miR-4472 | 27.718 | 34.250 | 7.997 | 9.537 | Down | 1.821 | 0.000 |
| hsa-miR-320b | 1.671 | 1.693 | 0.000 | 0.963 | Down | 1.804 | 0.024 |
| hsa-miR-128-1-5p | 1.376 | 1.792 | 0.000 | 1.060 | Down | 1.580 | 0.070 |
| hsa-miR-1267 | 1.376 | 1.294 | 0.000 | 0.963 | Down | 1.471 | 0.079 |
| hsa-miR-1306-3p | 1.573 | 1.294 | 1.086 | 0.000 | Down | 1.400 | 0.141 |
| hsa-miR-766-3p | 1.278 | 1.394 | 0.000 | 1.060 | Down | 1.334 | 0.141 |
| hsa-miR-129-1-3p | 0.983 | 2.091 | 0.000 | 1.252 | Down | 1.295 | 0.070 |
| hsa-miR-561-3p | 1.278 | 1.294 | 0.000 | 1.060 | Down | 1.279 | 0.196 |
| hsa-miR-29a-5p | 1.376 | 0.996 | 0.987 | 0.000 | Down | 1.264 | 0.163 |
| hsa-miR-1245a | 0.983 | 1.593 | 1.086 | 0.000 | Down | 1.246 | 0.196 |
| hsa-miR-1257 | 15.235 | 20.908 | 7.898 | 7.707 | Down | 1.212 | 0.000 |
| hsa-miR-1285-5p | 1.180 | 1.493 | 0.000 | 1.156 | Down | 1.209 | 0.141 |
| hsa-miR-375 | 1.180 | 1.593 | 0.000 | 1.252 | Down | 1.146 | 0.141 |
| hsa-miR-129-2-3p | 1.868 | 2.589 | 1.185 | 0.963 | Down | 1.053 | 0.126 |
| hsa-miR-708-3p | 1.180 | 1.195 | 0.000 | 1.156 | Down | 1.038 | 0.269 |
| hsa-miR-1254 | 1.769 | 1.394 | 1.580 | 0.000 | Down | 1.002 | 0.196 |

ESC1-N, ESC1 cultured under normoxic condition; ESC2-N, ESC2 cultured under normoxic condition;

ESC1-H, ESC1 cultured under hypoxic condition; ESC2-N, ESC2 cultured under hypoxic condition;
