# Supplementary material for: MiR-210-3p protects endometriotic cells from oxidative stress-induced cell cycle arrest by targeting BARD1
Source: Cell Death Dis. 2019 Feb 13;10(2):144. doi: 10.1038/s41419-019-1395-6 (PMC6374490; doi:10.1038/s41419-019-1395-6)
Supplement: Supplementary file 6 — Supplementary Table 5 [file 41419_2019_1395_MOESM6_ESM.docx]

**Supplementary Table 5**. List of Differentially Expression Genes identified by RNA-Sequencing in miR-210 overexpressing cells.

| gene | LV-CN | LV-210 | Up/Down | log2(Fold_change) | *P*-value | Description |
| --- | --- | --- | --- | --- | --- | --- |
| MIR210 | 0.000 | 20.584 | Up | Inf | 3.98E-11 | microRNA 210 |
| MIR21 | 2.656 | 20.638 | Up | 2.958 | 5.54E-05 | microRNA 21 |
| GDF15 | 24.225 | 63.564 | Up | 1.392 | 0 | growth differentiation factor 15 |
| DLL4 | 3.523 | 7.593 | Up | 1.108 | 0 | delta-like 4 (Drosophila) |
| ARID2 | 0.986 | 0.493 | Down | -1.000 | 5.10E-06 | AT rich interactive domain 2 (ARID, RFX-like) |
| TRIM59 | 2.549 | 1.260 | Down | -1.017 | 4.83E-07 | tripartite motif containing 59 |
| XIST | 1.296 | 0.639 | Down | -1.021 | 8.38E-16 | X inactive specific transcript (non-protein coding) |
| NCOA2 | 1.636 | 0.804 | Down | -1.024 | 3.22E-07 | nuclear receptor coactivator 2 |
| ENSG00000229807.5 | 1.263 | 0.621 | Down | -1.024 | 5.63E-16 | ENSG00000229807.5 |
| ZNF23 | 1.929 | 0.939 | Down | -1.038 | 4.90E-05 | zinc finger protein 23 |
| ANLN | 14.380 | 6.972 | Down | -1.044 | 4.32E-42 | anillin, actin binding protein |
| ARNTL2 | 2.389 | 1.141 | Down | -1.067 | 2.04E-12 | aryl hydrocarbon receptor nuclear translocator-like 2 |
| ARHGAP11A | 3.923 | 1.853 | Down | -1.083 | 5.07E-16 | Rho GTPase activating protein 11A |
| KIAA0895 | 1.098 | 0.516 | Down | -1.089 | 8.55E-05 | KIAA0895 |
| KIF11 | 2.913 | 1.366 | Down | -1.093 | 6.31E-11 | kinesin family member 11 |
| HMMR | 1.927 | 0.901 | Down | -1.097 | 2.89E-05 | hyaluronan-mediated motility receptor (RHAMM) |
| DLGAP5 | 3.617 | 1.675 | Down | -1.111 | 9.04E-09 | discs, large (Drosophila) homolog-associated protein 5 |
| CASC5 | 0.805 | 0.368 | Down | -1.127 | 1.57E-05 | cancer susceptibility candidate 5 |
| NRIP1 | 0.818 | 0.375 | Down | -1.127 | 1.57E-05 | nuclear receptor interacting protein 1 |
| ZNF367 | 2.859 | 1.242 | Down | -1.202 | 2.51E-09 | zinc finger protein 367 |
| MYOCD | 1.016 | 0.431 | Down | -1.236 | 4.16E-07 | myocardin |
| LZTS1 | 1.471 | 0.622 | Down | -1.242 | 1.03E-07 | leucine zipper, putative tumor suppressor 1 |
| POLQ | 0.545 | 0.226 | Down | -1.271 | 3.08E-05 | polymerase (DNA directed), theta |
| KIF14 | 0.885 | 0.360 | Down | -1.298 | 8.40E-07 | kinesin family member 14 |
| SLIT2 | 1.687 | 0.643 | Down | -1.391 | 3.19E-09 | slit homolog 2 (Drosophila) |
| RPS6KA6 | 0.590 | 0.224 | Down | -1.397 | 6.48E-06 | ribosomal protein S6 kinase, 90kDa, polypeptide 6 |
| DOPEY1 | 0.524 | 0.185 | Down | -1.502 | 9.43E-06 | dopey family member 1 |
| PLK4 | 1.133 | 0.390 | Down | -1.540 | 2.83E-06 | polo-like kinase 4 |
| BRIP1 | 0.976 | 0.321 | Down | -1.606 | 1.28E-10 | BRCA1 interacting protein C-terminal helicase 1 |
| BARD1 | 2.679 | 0.873 | Down | -1.618 | 1.63E-09 | BRCA1 associated RING domain 1 |
| KIF18A | 0.884 | 0.286 | Down | -1.627 | 6.60E-05 | kinesin family member 18A |

Inf, Infinity
