# Supplementary figures and images for: MiR-210-3p protects endometriotic cells from oxidative stress-induced cell cycle arrest by targeting BARD1
Source: Cell Death Dis. 2019 Feb 13;10(2):144. doi: 10.1038/s41419-019-1395-6 (PMC6374490; doi:10.1038/s41419-019-1395-6)

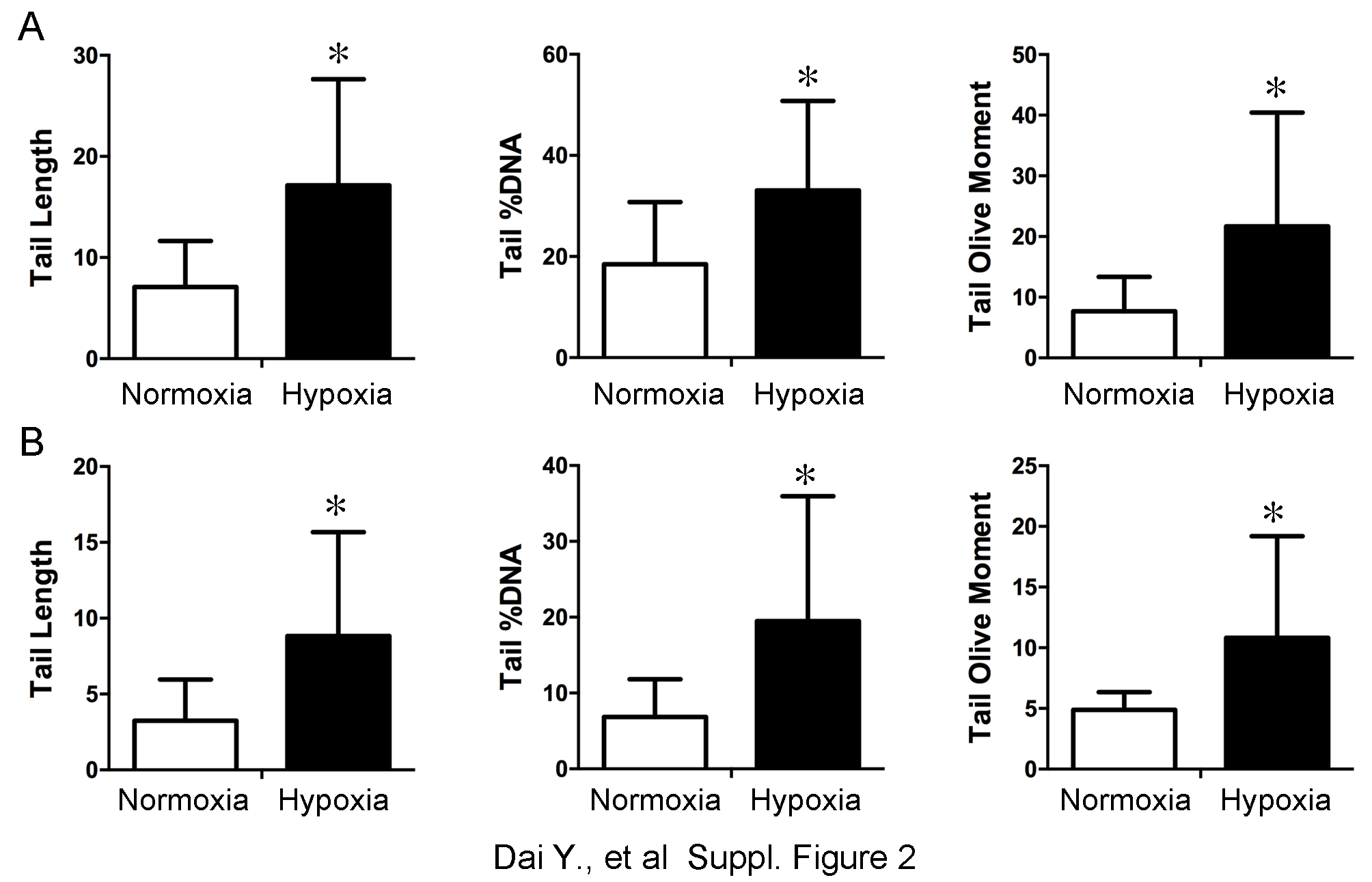

Supplement: Supplementary file 8 — Supplementary Figure 2 [file 41419_2019_1395_MOESM8_ESM.tif]

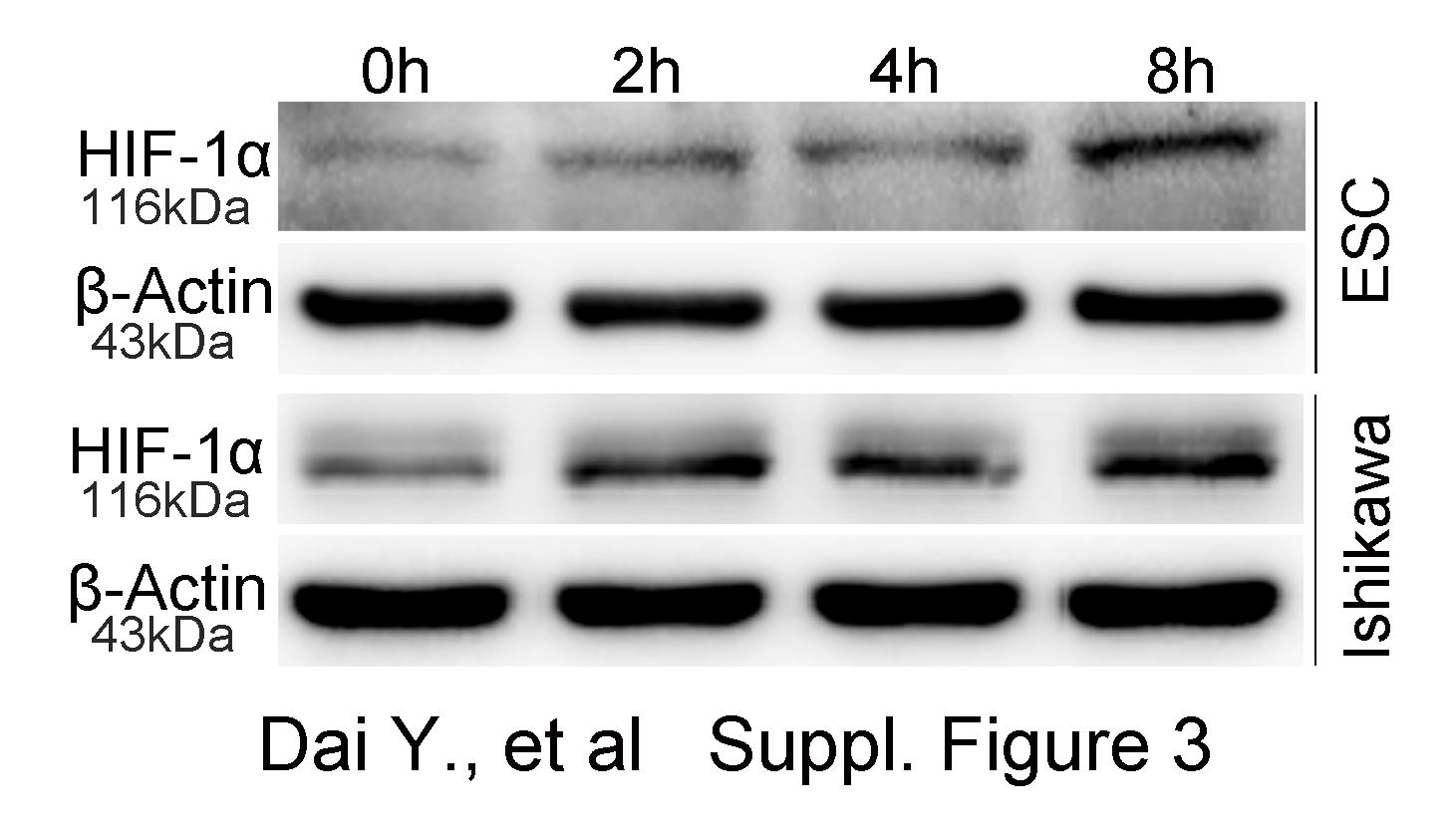

Supplement: Supplementary file 9 — Supplementary Figure 3 [file 41419_2019_1395_MOESM9_ESM.tif]

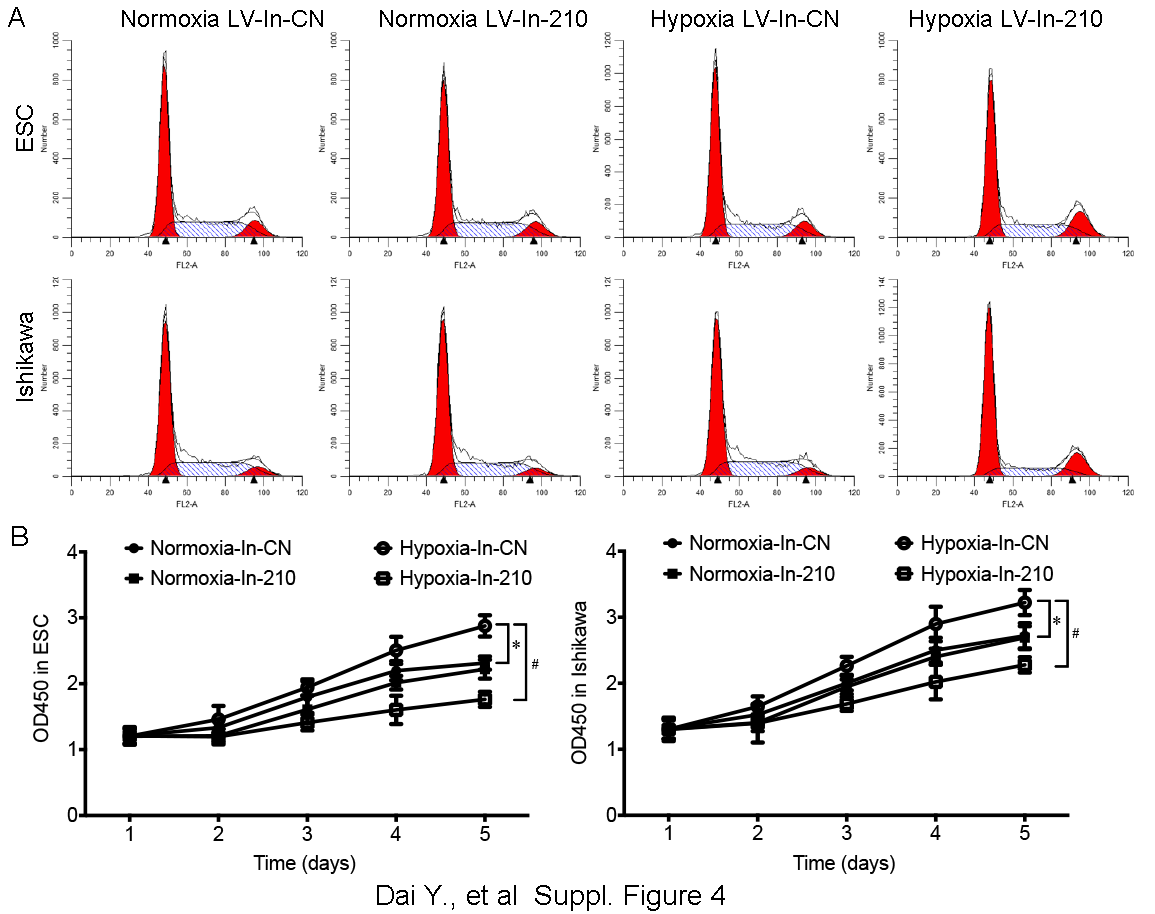

Supplement: Supplementary file 10 — Supplementary Figure 4 [file 41419_2019_1395_MOESM10_ESM.tif]

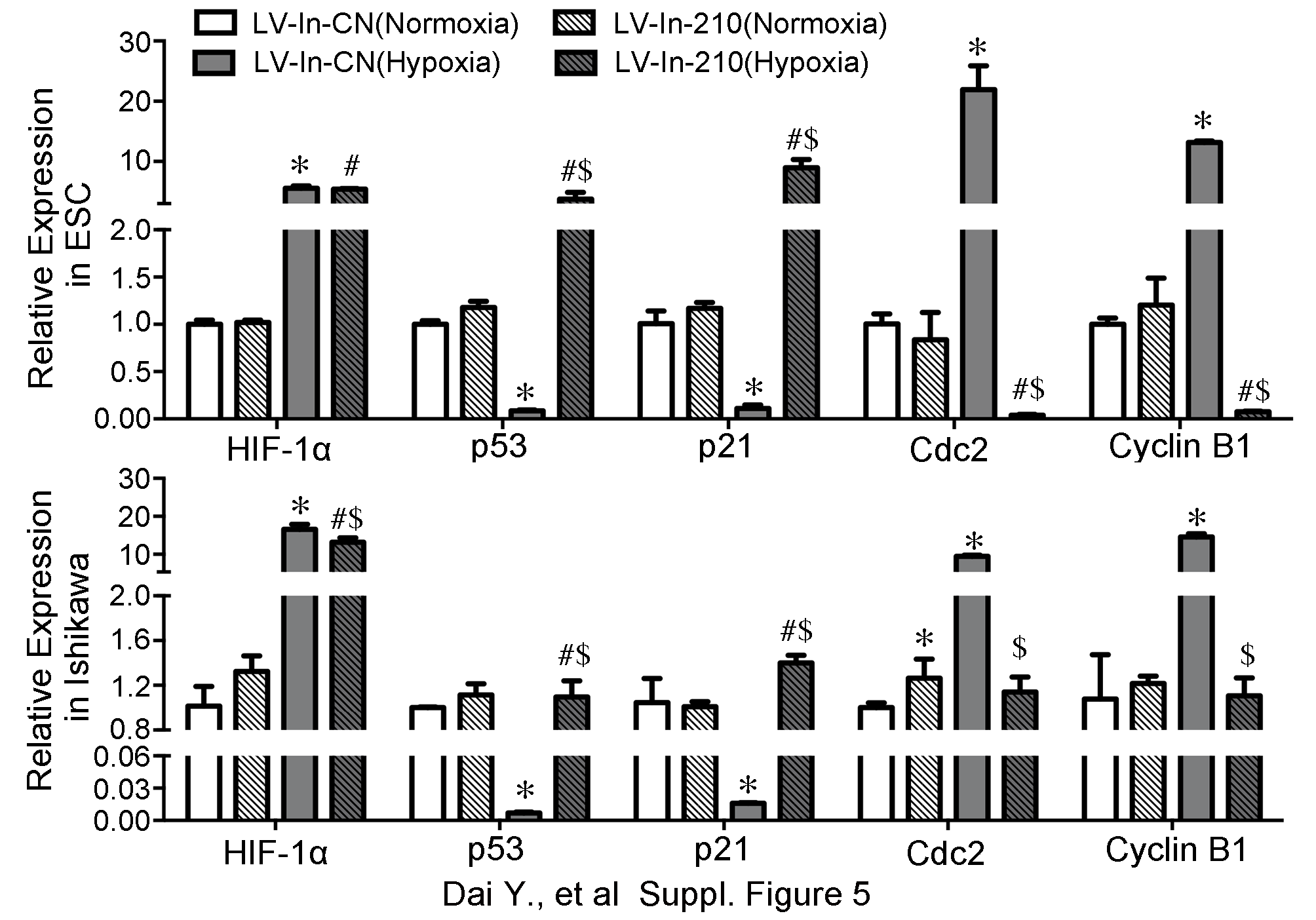

Supplement: Supplementary file 11 — Supplementary Figure 5 [file 41419_2019_1395_MOESM11_ESM.tif]

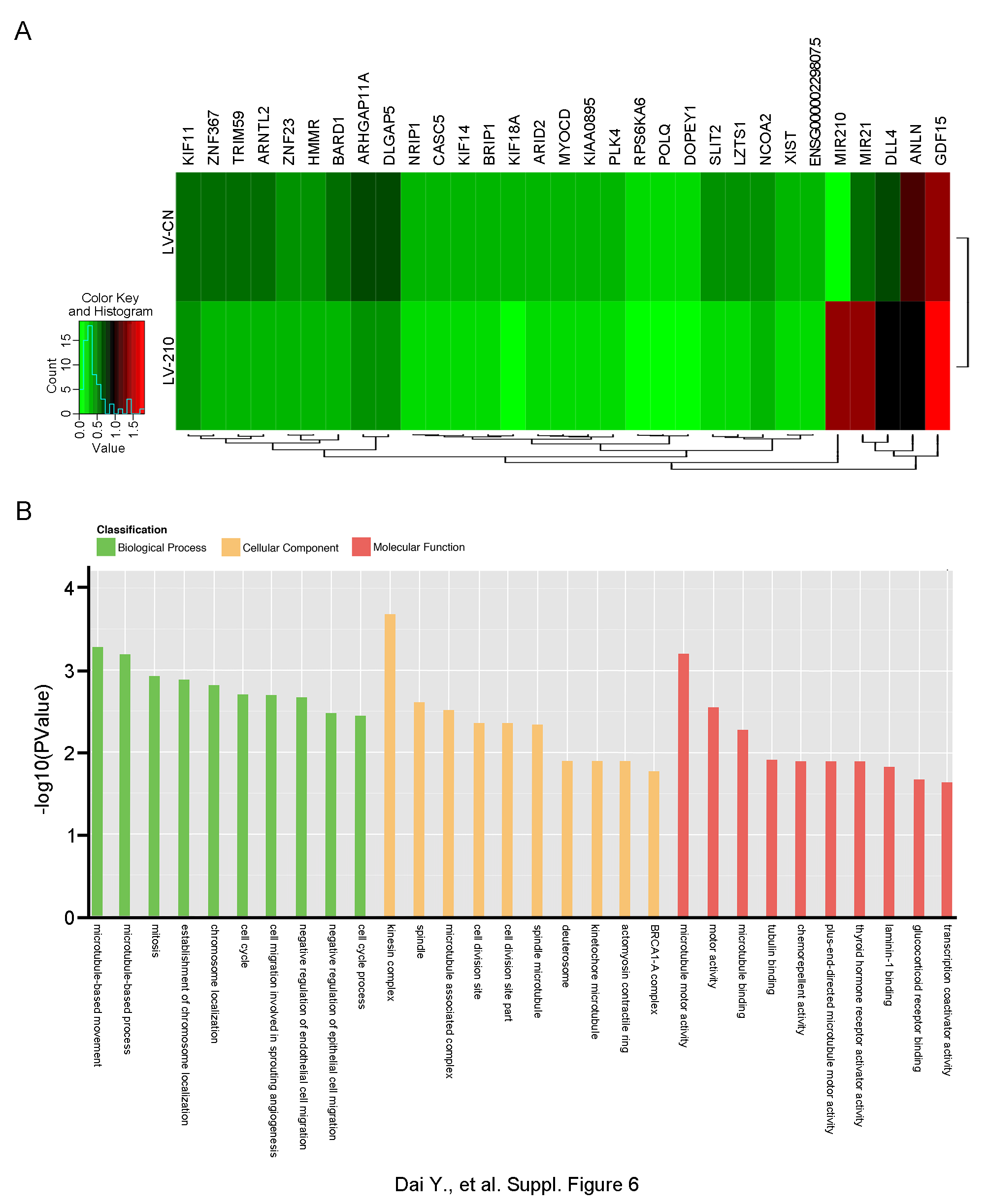

Supplement: Supplementary file 12 — Supplementary Figure 6 [file 41419_2019_1395_MOESM12_ESM.tif]

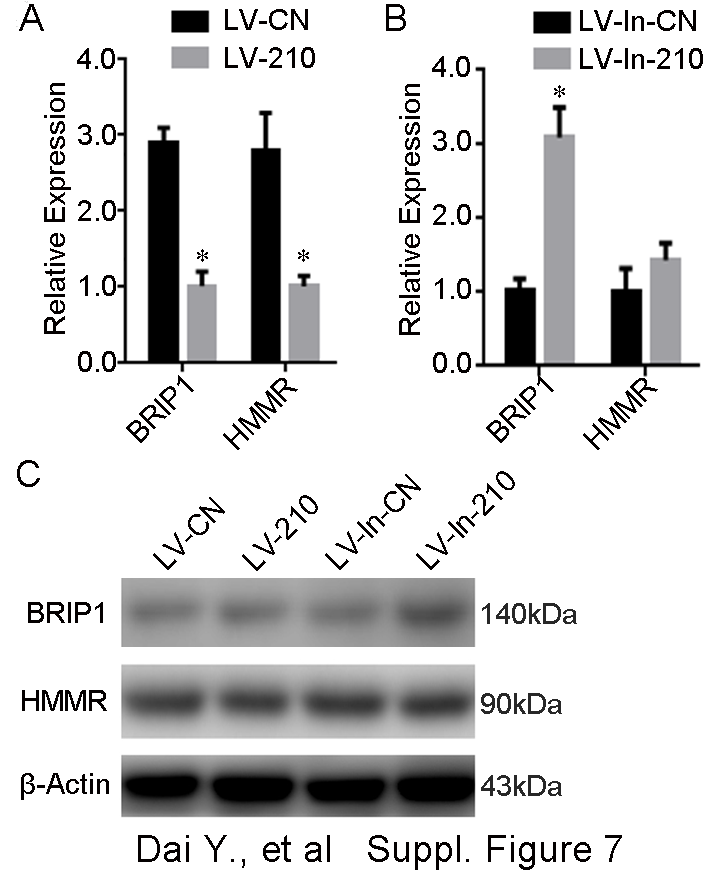

Supplement: Supplementary file 13 — Supplementary Figure 7 [file 41419_2019_1395_MOESM13_ESM.tif]

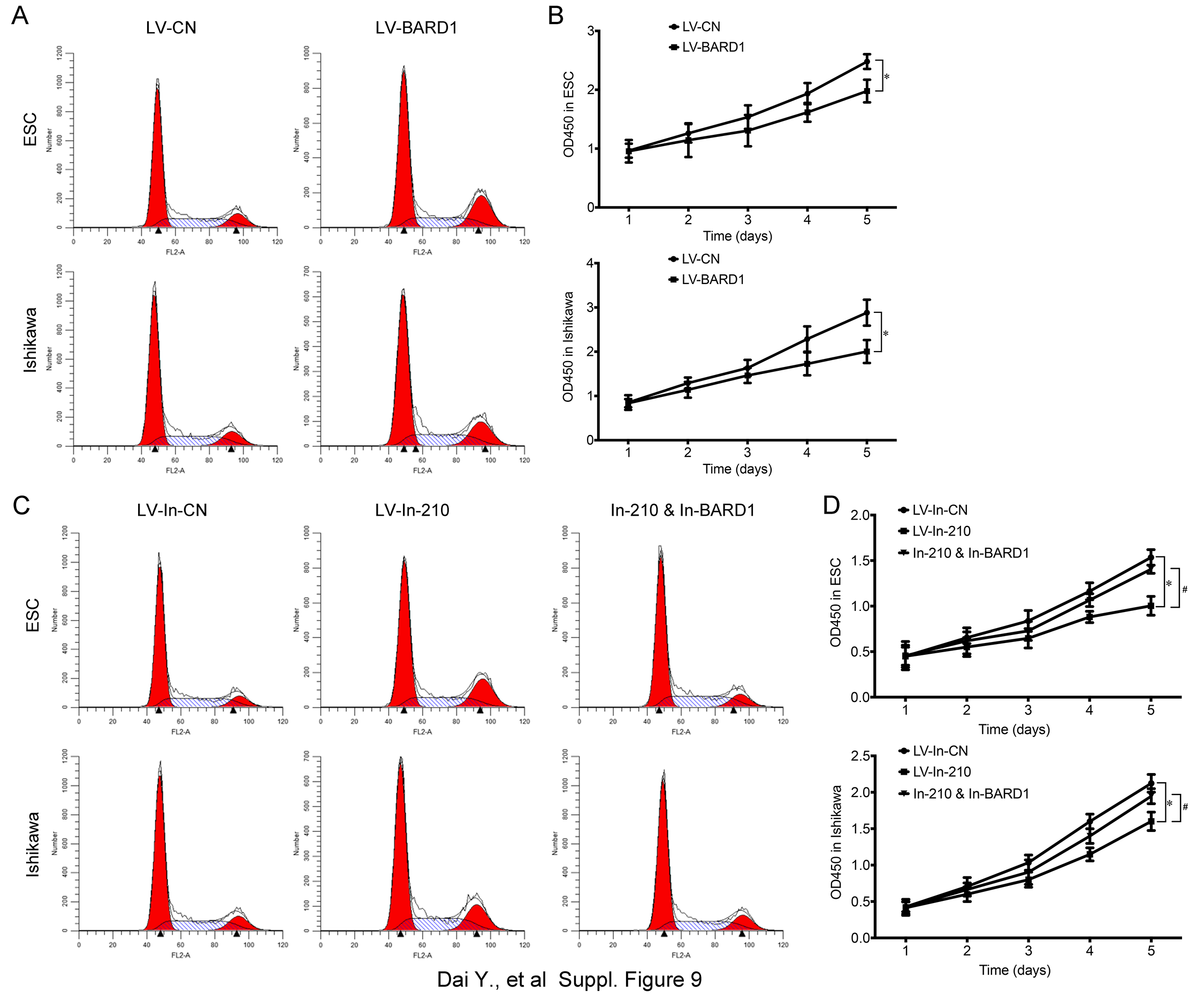

Supplement: Supplementary file 15 — Supplementary Figure 9 [file 41419_2019_1395_MOESM15_ESM.tif]
